# Stable iPSC-derived NKX2-1^+^ lung bud tip progenitor organoids give rise to airway and alveolar cell types

**DOI:** 10.1242/dev.200693

**Published:** 2022-08-30

**Authors:** Renee F. C. Hein, Ansley S. Conchola, Alexis S. Fine, Zhiwei Xiao, Tristan Frum, Lindy K. Brastrom, Mayowa A. Akinwale, Charlie J. Childs, Yu-Hwai Tsai, Emily M. Holloway, Sha Huang, John Mahoney, Idse Heemskerk, Jason R. Spence

**Affiliations:** 1 Department of Cell and Developmental Biology, University of Michigan Medical School, Ann Arbor, MI 48109, USA; 2 Department of Computational Medicine and Bioinformatics, University of Michigan Medical School, Ann Arbor, MI 48109, USA; 3 Program in Cell and Molecular Biology, University of Michigan Medical School, Ann Arbor, MI 48109, USA; 4 Department of Internal Medicine, University of Michigan Medical School, Ann Arbor, MI 48109, USA; 5 Therapeutics Lab, Cystic Fibrosis Foundation, Lexington, MA 02421, USA; 6 Department of Biomedical Engineering Medicine, University of Michigan Medical School, Ann Arbor, MI 48109, USA

**Keywords:** Lung organoid, Bud tip progenitor, Human pluripotent stem cell, Single cell RNA sequencing

## Abstract

Bud tip progenitors (BTPs) in the developing lung give rise to all epithelial cell types found in the airways and alveoli. This work aimed to develop an iPSC organoid model enriched with NKX2-1^+^ BTP-like cells. Building on previous studies, we optimized a directed differentiation paradigm to generate spheroids with more robust NKX2-1 expression. Spheroids were expanded into organoids that possessed NKX2-1^+^/CPM^+^ BTP-like cells, which increased in number over time. Single cell RNA-sequencing analysis revealed a high degree of transcriptional similarity between induced BTPs (iBTPs) and *in vivo* BTPs. Using FACS, iBTPs were purified and expanded as induced bud tip progenitor organoids (iBTOs), which maintained an enriched population of bud tip progenitors. When iBTOs were directed to differentiate into airway or alveolar cell types using well-established methods, they gave rise to organoids composed of organized airway or alveolar epithelium, respectively. Collectively, iBTOs are transcriptionally and functionally similar to *in vivo* BTPs*,* providing an important model for studying human lung development and differentiation.

## Abstract (precis)

**Summary:** iPSC-derived lung bud tip progenitors emerge in organoid culture, can be isolated and expanded, are transcriptionally similar to primary bud tip progenitors, and can differentiate into airway or alveolar organoids.

## INTRODUCTION

Advances in directed differentiation methods have led to the development of numerous embryonic or induced pluripotent stem cell (iPSC)-derived cell and organoid models of the airway and alveoli, which have enhanced our ability to model human lung development and disease (Wang et al., 2007; Mou et al., 2012; Gotoh et al., 2014; Huang et al., 2014; Dye et al., 2015, 2016; Konishi et al., 2016; Chen et al., 2017; McCauley et al., 2017; Yamamoto et al., 2017, 2020; Hawkins et al., 2017; Miller et al., 2018, 2019; Tamò et al., 2018; de Carvalho et al., 2019; Jacob et al., 2019; Leibel et al., 2020). Airway and alveolar cell types in mice and humans are derived from a common, developmentally transient progenitor population, called bud tip progenitors, which reside at the tips of the branching tree-like network of tubes that make up the lung epithelium (Serra et al., 1994; Perl et al., 2005; Abler et al., 2008; Goss et al., 2009; Rawlins et al., 2009; Rockich et al., 2013; Alanis et al., 2014; Nikolić et al., 2017; Miller et al., 2018).

Bud tip progenitors obtained from the human fetal lung and grown as organoids serve as a useful tool for studying the mechanisms responsible for bud tip progenitor cell maintenance and differentiation into airway and alveolar cell types (Nikolić et al., 2017; Miller et al., 2018, 2020; Conway et al., 2020; Sun et al., 2020). Despite this progress, organoids derived from fetal tissue are not broadly accessible to the research community and are associated with ethical and regulatory challenges, emphasizing the importance of iPSC-derived lung models. Although we and others have made some progress in developing bud tip progenitor cell-like models from iPSCs (Chen et al., 2017; Miller et al., 2018), new technologies such as single cell RNA-sequencing (scRNA-seq) and single cell lineage tracing have highlighted off-target cell types and unexpected plasticity in iPSC-derived cultures, where cells appear to be committed to a specific cell type or lineage but subsequently change fate (Little et al., 2019; Hurley et al., 2020). This concept of cellular plasticity within the lung has also been demonstrated *in vivo*, where hyperactive WNT signaling in lung progenitors was shown to cause differentiation of intestinal cells in transgenic mouse embryos (Okubo and Hogan, 2004). Therefore, a challenge in the field, addressed in the current work, is to develop a long-lived and transcriptionally stable bud tip progenitor-like model from iPSCs.

Single cell RNA-sequencing technologies have also made it possible to benchmark iPSC-derived cultures against primary tissue to compare transcriptional similarity and to accurately catalogue the diversity of on-target or off-target cell types observed *in vitro* (Hawkins et al., 2017, 2021; McCauley et al., 2018; Holloway et al., 2020; Hor et al., 2020; Yu et al., 2020). As iPSC-derived cultures are known to be plastic and iPSC differentiation is not 100% efficient, benchmarking has become an important step towards understanding the full complement of cells present in a culture. This study therefore also sought to benchmark iPSC-derived bud tip progenitor organoids to interrogate the diversity of cell types in culture and the similarity to primary bud tip progenitor organoids from the fetal lung.

Here, we have optimized an iPSC directed differentiation paradigm to generate self-organizing 3D spheroids with robust NKX2-1 expression. Expansion of NKX2-1^+^ cells in bud tip progenitor medium over 3-17 weeks gave rise to heterogenous organoids that contained NKX2-1^+^ bud tip progenitor-like cells co-expressing markers of human bud tip progenitors, including SOX9, SOX2 and the cell surface marker CPM (Yamamoto et al., 2020). Using an NKX2-1 reporter iPSC line along with CPM to quantitatively assess cultures via flow cytometry, we observed that bud tip progenitor-like cells expanded over subsequent weeks in culture. FACS isolation and further culture allowed for the expansion of NKX2-1^+^/CPM^+^ cells as bud tip progenitor-like organoids (iBTOs) that maintained ∼80% NKX2-1^+^/CPM^+^ cells for at least 8 weeks. scRNA-seq analysis of bud tip progenitor cells from unsorted organoids or after FACS-enrichment revealed a high degree of transcriptional similarity to primary bud tip progenitor organoids, as well as a shared transcriptional signature with *in vivo* bud tip progenitors. In addition, scRNA-seq from iBTOs that have spent less (3 weeks) or more (10 weeks) time in culture suggest that induced bud tip progenitors become more transcriptionally similar to native bud tip progenitors as they age. Finally, we used well-established methods to direct differentiation of iBTOs into organoids composed of airway epithelium (including basal, secretory, ciliated, goblet and neuroendocrine cells) or alveolar type II (AT2) cells (Miller et al., 2020; Jacob et al., 2017). Collectively, this study describes a robust method to generate bud tip progenitor-like cells from iPSCs that closely resemble organoids derived from primary tissue. This model can be readily used to study lung development and illustrates a proof-of-concept for cellular engineering and cell therapy.

## RESULTS

### Lung spheroids are optimized for NXK2-1 expression but remain heterogenous

NKX2-1 is the earliest marker during lung epithelial specification (Lazzaro et al., 1991) and loss of NKX2-1 leads to lung agenesis (Minoo et al., 1995, 1999; Little et al., 2019; Kuwahara et al., 2020). We therefore sought to build on a previously published method to generate iPSC-derived foregut spheroids (Dye et al., 2015) by optimizing endoderm induction efficiency and foregut spheroid development in order to improve NKX2-1 expression.

We began by testing conditions to improve definitive endoderm (DE) differentiation efficiency and reproducibility. DE induction of varying efficiencies is achieved by using activin A (ACTA) ligand (Kubo et al., 2004; D'Amour et al., 2006; Spence et al., 2011) with WNT and BMP signaling playing a synergistic role during the initial stages of DE specification (Gadue et al., 2006; Green et al., 2011; Loh et al., 2014; Matsuno et al., 2016; Rankin et al., 2018; Heemskerk et al., 2019). Therefore, we tested combinations of ACTA alongside the small molecule WNT activator CHIR99021 (CHIR) or BMP4 on the first day of a 3-day ACTA differentiation culture. Using flow cytometry with a SOX17-tdTomato and SOX2-mCITRINE hESC reporter line (Martyn et al., 2018) to quantitate cell composition, we observed that ACTA alone induced 48% SOX17^+^ cells, while the addition of CHIR or BMP4 both enhanced DE differentiation, leading to 96% SOX17^+^ cells or 87% SOX17^+^ cells, respectively (Fig. S1A). Addition of CHIR and BMP4 together led to 71% SOX17^+^ cell induction (Fig. S1A). DE cultures from an additional cell line were co-stained with SOX17 and FOXA2 to confirm definitive endoderm cell identity (Fig. S1B). We observed that near-pure SOX17^+^ cultures obtained via ACTA and CHIR failed to give rise to self-organizing 3D spheroids, consistent with published data showing that self-organizing foregut and hindgut organoids consist of both epithelium and mesenchymal lineages (Spence et al., 2011; Dye et al., 2015, 2016). Therefore, ACTA and BMP4 were used for subsequent experiments.

After DE specification, monolayers were directed into 3D foregut endoderm spheroids by combining a method that efficiently induces ventral foregut endoderm competent to be specified as lung (Rankin et al., 2016) alongside methods that induce 3D self-organization (Spence et al., 2011; Dye et al., 2015). This included BMP inhibition via noggin (NOG), FGF4 and CHIR (required for 3D spheroid formation) for 3 days plus all-trans retinoic acid (ATRA) on the last day (Fig. S1C,D). After 3 days, spheroids were collected and suspended in Matrigel and treated for 3 additional days with low BMP4 as well as WNT3A and RSPO1, which together stimulate WNT signaling (Shu et al., 2005; Goss et al., 2009; Harris-Johnson et al., 2009; Domyan et al., 2011; Jacobs et al., 2012; Miller et al., 2012; Serra et al., 2017). WNT activation by combined RSPO and WNT or by CHIR resulted in comparable expression levels of foregut and hindgut markers (Fig. S1E); however, CHIR is a GSK3β inhibitor and can have non-WNT mediated effects, so RSPO and WNT were used as more specific activators of WNT signaling.

**Fig. 1.**
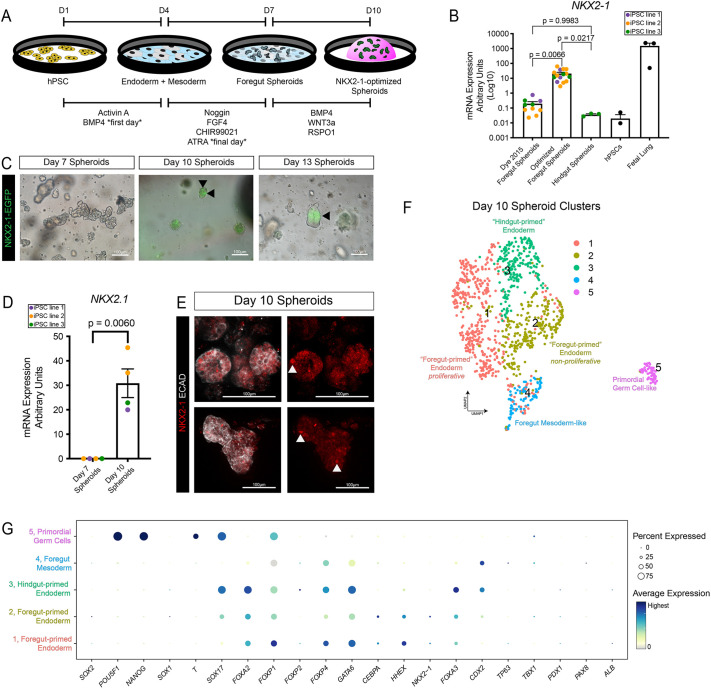
**Optimization of lung spheroids for NKX2-1 expression.** (A) Schematic displaying the directed differentiation protocol from hPSCs to NKX2-1-optimized spheroids. (B) qRT-PCR data comparing *NKX2-1* expression in previously published foregut spheroids (Dye et al., 2015) with optimized foregut spheroids (this protocol) and hindgut spheroids (Spence et al., 2011). hPSCs and whole fetal lung are also included as references. Each colored dot represents a technical replicate from one or more experiments with a unique iPSC line (purple, WTC11; orange, iPSC17 WT 7B2; green, iPSC line 72.3). Data are mean±s.e.m. Statistical tests used were an ordinary one-way ANOVA followed by Tukey's multiple comparison test. (C) Representative reporter expression for NKX2-1-EGFP on day 7, 10 or 13 spheroids. (D) qRT-PCR data comparing *NKX2-1* expression in spheroids collected on day 7 or day 10 (see A). Each colored dot represents the result of an independent experiment with a unique iPSC line (purple, WTC11; orange, iPSC17 WT 7B2; green, iPSC line 72.3). Data are mean±s.e.m. The statistical test used was an unpaired Welch's one-tailed *t*-test. (E) Maximum intensity projection of a whole-mount immunofluorescence confocal *z*-series stained for the pan-epithelial marker ECAD and the lung epithelial marker NKX2-1 on day 10 spheroids. (F) UMAP cluster plot of scRNA-seq data from day 10 spheroids (*n*=1 batch, ∼100 spheroids). Each dot represents a single cell and cells were computationally clustered based on transcriptional similarities. The plot is colored and numbered by cluster. Cell-type labels for each cluster are based on expression of canonical cell-type markers displayed in the dot plot in G or Fig. S1G. (G) Dot plot of cell lineage genes in each cluster of the UMAP plot in F. The dot size represents the percentage of cells expressing the gene in the corresponding cluster; the dot color indicates log-normalized expression level of the gene.

At the end of the 9-day directed differentiation (Fig. 1A), spheroids were analyzed for *NKX2-1* expression via qRT-PCR (on day 10) (Fig. 1B). When this optimized method was directly compared with spheroids generated using previously published foregut/lung spheroid protocols (Dye et al., 2015; Miller et al., 2019), NKX2-1-optimized foregut spheroids expressed ∼100-fold more *NKX2-1* (Fig. 1B). Undifferentiated iPSCs and hindgut spheroids were included as controls, both of which had very low *NKX2-1*, and fetal lung was used as a positive control. By analyzing EGFP expression using a NKX2-1-EGFP reporter cell line, EGFP was not detected at day 7, whereas a low level of ubiquitous expression with scattered NKX2-1^HI^ cells could be detected starting on day 10, and expression was localized to specific regions by day 13 (Fig. 1C). *NKX2-1* induction between day 7 and day 10 was confirmed by qRT-PCR (Fig. 1D), and whole-mount immunofluorescence of day 10 spheroids correlated with reporter expression, with individual cells expressing high levels of NKX2-1 protein (Fig. 1E, arrowheads).

When day 10 spheroids were analyzed by scRNA-seq, *NKX2-1* was also observed in a subset of cells (Fig. 1F,G), revealing heterogeneity within the foregut spheroids and supporting EGFP reporter expression. Spheroids contained two clusters (clusters 1 and 2) of lung-fated ventral foregut endoderm-like cells expressing *NKX2-1*, *FOXA2*, *FOXP1*, *FOXP4* and *HHEX* (Bogue et al., 1998; Shu et al., 2007; Spence et al., 2009; Kearns et al., 2013; Davenport et al., 2016; Li et al., 2016). Clusters and cells were largely negative for other foregut lineage markers, including *TP63*, *TBX1*, *PDX1*, *PAX8* and *ALB*; however, there was a clear population of cells that express *FOXA2*, *FOXA3*, *SOX17* and *CDX2* (cluster 3), which we refer to as ‘hindgut-primed’ endoderm. Additionally, we observed a cluster of *CDX2*^+^/*HAND1*^+^/*ISL1*^+^/*BMP4*^+^/*FOXF1*^+^/*LEF1^+^* (cluster 4) foregut mesoderm-like cells (Han et al., 2020) and a small population of cells (cluster 5) expressing markers indicative of primordial germ cells, including *POU5F1*, *NANOG*, *T* (*TBXT*), *SOX17*, *NANOS3* and *TFAP2C* (Davenport et al., 2016; Jo et al., 2022) (Fig. 1G, Fig. S1G). Comparing optimized foregut spheroids to previously published methods (Dye et al., 2015; Miller et al., 2019) by qRT-PCR, we observed that the hindgut endoderm marker *CDX2* was not statistically different, but the early foregut and dorsal foregut endoderm marker *SOX2* (Que et al., 2007) was reduced with the optimized method (Fig. S1F), which suggests previous methods are competent to generate foregut that is relatively immature or more dorsal rather than ventral. Taken together, our data show that optimized foregut spheroids have much higher levels of NKX2-1 when compared with previous methods, but they are still heterogeneous with distinct populations of lung-fated cells and hindgut-fated cells.

### iPSC-derived bud tip progenitors emerge over time

**Fig. 2.**
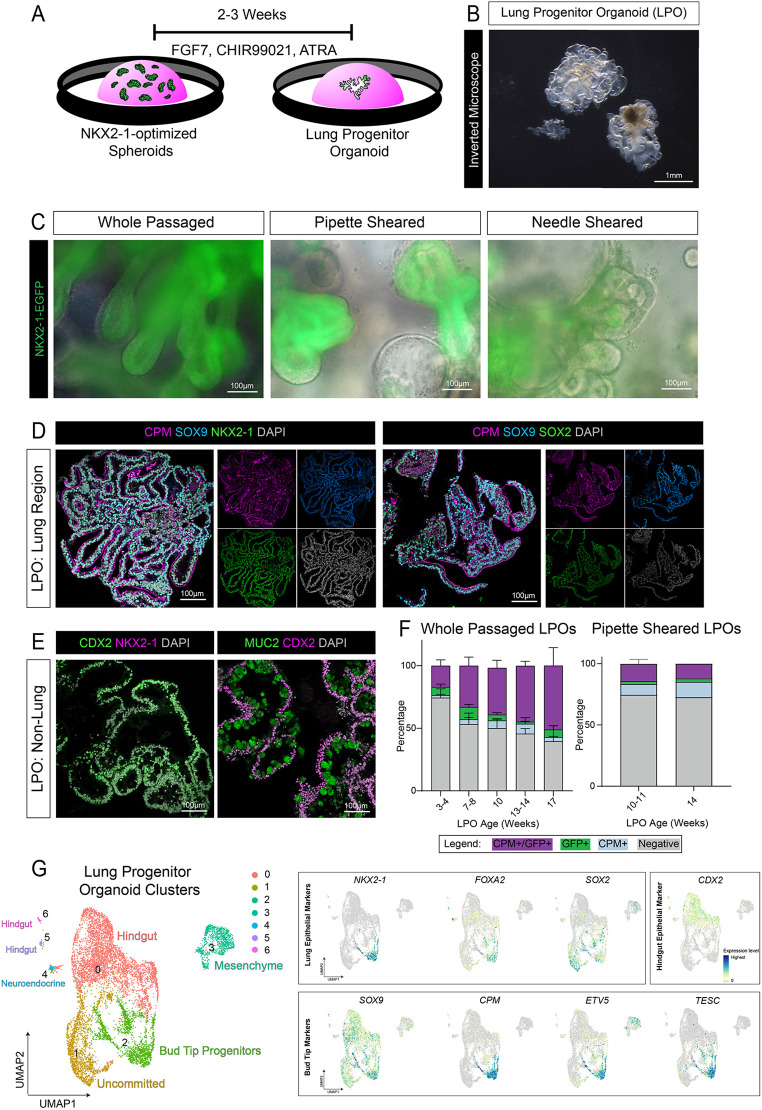
**iPSC-derived bud tip progenitors emerge over time in LPOs.** (A) Schematic displaying the lung progenitor organoid (LPO) expansion protocol from NKX2-1-optimized spheroids. LPOs form after 2-3 weeks in culture. (B) Bright-field image of 6-week LPOs on an inverted microscope. (C) Representative NKX2-1-EGFP reporter images of 10- to 11-week LPOs, passaged whole or sheared (pipette and needle). (D) Immunofluorescence staining of paraffin wax-embedded sections of the lung-like regions of 12-week LPOs for bud tip progenitor markers CPM and SOX9, and the lung epithelial markers NKX2-1 (left) or SOX2 (right). (E) Immunofluorescence staining on paraffin wax-embedded sections for the intestinal epithelial marker CDX2, the intestinal goblet cell marker MUC2 and the lung epithelial marker NKX2-1 on non-lung regions of 12-week LPOs. (F) FACS quantification of NKX2-1-EGFP^+^/CPM^+^ cells in whole passaged or pipette-sheared LPOs in aggregate time course (3-17 weeks) from the NKX2-1-EGFP reporter cell line (iPSC17 WT 7B2). Percentages of live cells expressing neither marker (negative, gray) or each separate marker (CPM^+^ only, blue; EGFP^+^ only, green), or dual-expressing cells (CPM^+^/EGFP^+^, purple) are reported as mean±s.e.m. for 3-7 replicates per time point. (G) UMAP cluster plot of scRNA-seq data from LPOs (*n*=2 biological replicates for 3- and 6-week timepoints, *n*=1 for 10-week timepoint). Each dot represents a single cell and cells were computationally clustered based on transcriptional similarities. The plot is colored and numbered by cluster. Cell-type labels for each cluster are based on expression of canonical cell-type markers displayed in the heat map in Fig. S3 and the dot plot and feature plots in G and Fig. S2G. Feature plots corresponding to the LPO cluster plot and displaying canonical bud tip progenitor markers (*SOX9*, *CPM*, *ETV5* and *TESC*) (Miller et al., 2018, 2020; Yamamoto et al., 2020), lung epithelial markers (*NKX2-1*, *FOXA2* and *SOX2*) and a hindgut epithelial marker (*CDX2*). The color of each dot in the feature plot indicates log-normalized expression level of the labeled gene in the represented cell.

Once NKX2-1^+^ spheroids were formed, we asked how efficiently these spheroids would give rise to bud tip progenitor (BTP)-like cells as spheroids expanded into larger organoid structures. Our previous studies have shown that ‘3 Factor (3F) medium’ possessing FGF7, CHIR99021 and ATRA expands primary BTPs derived from the fetal lung and SOX2^+^/SOX9^+^ iPSC-derived BTP-like cells; however, efficiency and culture over prolonged periods of time were not assessed (Miller et al., 2018). After inducing NKX2-1-optimized spheroids on day 10, medium was switched to 3F BTP medium (Fig. 2A). Spheroids were maintained in 3F for several weeks, where they expanded into complex, branching structures termed ‘lung progenitor organoids (LPOs)’, similar to what we have observed previously (Miller et al., 2018) (Fig. 2B). LPOs were passaged every 2-3 weeks as either intact organoids with minimum fragmentation (whole passaged) or were sheared by being drawn through a hypodermic needle or pipette, which is a standard method for passaging BTP organoids derived from primary tissue (Miller et al., 2018, 2020; Hein et al., 2022). Using an NKX2-1-EGFP reporter iPSC line to compare retention of lung identity after various passaging methods, whole passaged organoids maintained robust EGFP^+^ reporter expression while fragmenting organoids ultimately led to a loss of NKX2-1-EGFP^+^ cells (Fig. 2C, quantified in Fig. 2F). Based on the maintenance of NKX2-1-EGFP expression, we therefore chose to use whole passaged LPOs for our remaining experiments.

We evaluated the presence of induced bud tip progenitor (iBTP) cells within whole passaged LPOs by immunofluorescence of paraffin wax-embedded sections and whole-mount organoids (Fig. 2D, Fig. S2A). LPOs possessed regions of NKX2-1-expressing cells, which co-expressed the BTP markers CPM, SOX9 and SOX2 (Nikolić et al., 2017; Miller et al., 2018; Yamamoto et al., 2020) (Fig. 2D, Fig. S2A, Movie 1). LPOs also contained sub-regions of distinct NKX2-1^−^/CDX2^+^ cells, with many expressing MUC2, indicative of goblet-like intestinal cells, and smaller regions of NKX2-1^−^/CDX2^−^ cells (Fig. 2E, Fig. S2B, Movie 2).

We quantitatively assessed EGFP^+^/CPM^+^ cells in LPOs grown for 3-17 weeks in 3F medium using fluorescence-activated cell sorting (FACS) (Fig. 2F, Fig. S2C). We observed an increase in EGFP^+^/CPM^+^ cells in culture over time (Fig. 2F, purple bars). At 3.5 weeks, 17% of cells expressed CPM and EGFP, in contrast to 17 weeks, where 51% of cells in culture were EGFP^+^/CPM^+^ (Fig. 2F). A small proportion (<10%) of cells were singly positive for EGFP (green) or CPM (blue) at any timepoint, and a population of double-negative cells was observed at all times (Fig. 2F), suggesting that some heterogeneity is maintained in LPOs. We also investigated LPOs derived from two additional non-reporter iPSC lines, using only CPM to quantify iBTPs. We observed a similar increase of iBTPs in culture over time, with 19% CPM^+^ cells at 3.5 weeks and 42% CPM^+^ cells by 17 weeks (Fig. S2D, left). Finally, organoids derived from previously published lung organoid protocols (Dye et al., 2015; Miller et al., 2019) contained approximately 0.5-6% CPM^+^ cells at any timepoint examined (Fig. S2D, right). The increase in CPM^+^ cells over time suggests that optimized culture conditions promote the emergence and selection of iBTPs.

To further interrogate the heterogeneity and complexity of the LPOs, we performed scRNA-seq on whole passaged LPOs at 3, 6 and 10 weeks (Fig. 2G). We identified one cluster with robust levels of *NKX2-1* and BTP gene expression (cluster 2), several clusters enriched for hindgut markers (clusters 0, 5 and 6), a mesenchymal cluster (cluster 3), a neuroendocrine-like cluster (cluster 4) and a cluster of unknown/uncommitted cells (cluster 1) (Fig. 2G, Figs S2G, S3). The proportion of cells in the BTP cluster (cluster 2) increased over time, while mesenchyme (cluster 3) was depleted over time and hindgut cells (clusters 0, 5, 6) were persistent (Fig. S2E,F). Together these data support FACS data suggesting that iBTPs continue to expand within LPOs over time and identify contaminating lineages that persist.

To assess the proliferation of the cultures, we quantified KI67 expression within each cluster and sample from the LPO scRNA-seq data (Fig. S2H). *KI67*^+^ cells were present in every cluster; however, the hindgut (clusters 0, 5 and 6), BTP (cluster 2) and mesenchymal (cluster 3) clusters accounted for 92.24% of *KI67*^+^ cells within the culture compared with 7.76% from the uncommitted (cluster 1) and neuroendocrine (cluster 4) clusters (Fig. S2H, top). Contribution of *KI67*^+^ cells to each cluster by sample was also calculated and normalized to the number of cells within each sample (Fig. S2H, bottom). The contribution of samples to the *KI67*^+^ cells within clusters was varied. Between the 3-week samples, there were proliferating cells that contributed to all clusters; however, there was a significant contribution of this early timepoint to the uncommitted cluster (cluster 1), which comprised 87.11% *KI67*^+^ cells. The 6-week samples also contributed *KI67*^+^ cells to all the clusters, with the most significant contributions to hindgut (clusters 0, 5 and 6) and the mesenchyme (cluster 3). The data suggest that by 6 weeks any remaining contaminating cell types (i.e. *CDX2*^+^ intestinal cells) are most proliferative, while the earlier cultures are altogether generally proliferative. Finally, the 10-week sample contributed a significant proportion of proliferating cells to the BTP cluster (cluster 2; 39.14% of *KI67*^+^ cells), the neuroendocrine cluster (cluster 4) and the two smaller hindgut clusters (clusters 5 and 6) (Fig. S2H, top). Importantly, all samples contributed to the BTP cluster, with a robust contribution from the 10-week sample (Fig. S2H, top).

Cell death in LPOs, evaluated by Hematoxylin and Eosin, and cleaved caspase 3 (CCAS3) stains, was evident at all stages of growth and did not appear to increase with time or localize to contaminating CDX2^+^ regions; however, localization of CCAS3 staining transitioned from individual cells at 3 weeks to luminal regions by 17 weeks in culture (Fig. S2I). These data suggest that the increasing number of iBTP cells observed is not likely due to death of contaminating cell types.

### iPSC-derived bud tip progenitors can be isolated, expanded and maintained long-term

**Fig. 3.**
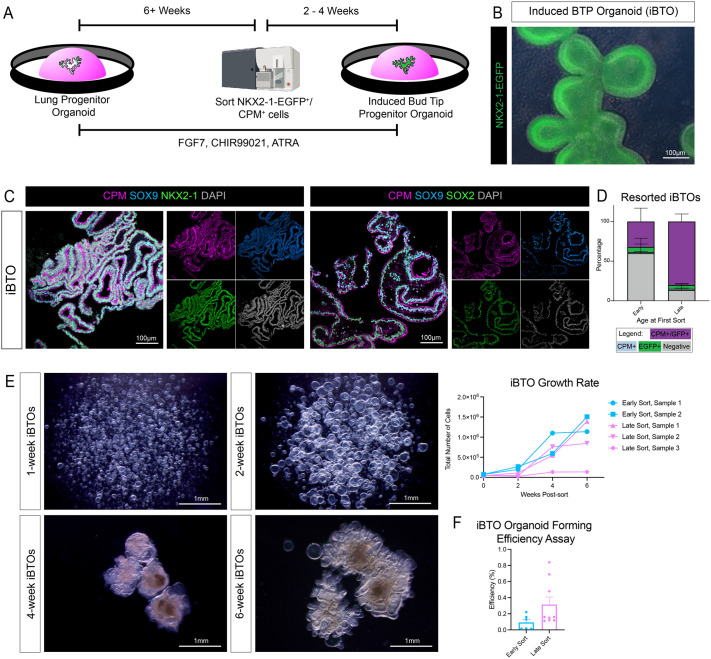
**iPSC-derived bud tip progenitors can be isolated and expanded in the long term**. (A) Schematic displaying isolation and expansion of induced bud tip progenitor organoids (iBTOs). LPOs are maintained in 3F medium and whole passaged for at least 6 weeks, then dissociated for FACS. iBTPs are isolated using CPM^+^ expression with or without NKX2-1-EGFP^+^ reporter expression and replated as isolated iBTPs. iBTPs re-form to iBTOs over 2-4 weeks, are maintained in 3F medium and whole passaged. Schematic created using Biorender.com. (B) Representative NKX2-1-EGFP reporter image of 3-week iBTOs. (C) Immunofluorescence staining on paraffin wax-embedded sections of nearly homogenous 4-week iBTOs for bud tip progenitor markers CPM and SOX9, and the lung epithelial markers NKX2-1 (left panels) or SOX2 (right panels). (D) FACS quantification of NKX2-1-EGFP^+^/CPM^+^ cells in iBTOs from 3-week sorted LPOs (early) or 8-17 week sorted LPOs (late) from the NKX2-1-EGFP reporter cell line (iPSC17 WT 7B2). iBTOs were analyzed 7-8 weeks after iBTP purification from LPOs. Percentages of live cells expressing neither marker (negative, gray) and each separate marker (CPM^+^ only, blue; EGFP^+^ only, green), or dual-expressing cells (CPM^+^/EGFP^+^, purple) are reported as mean±s.e.m. for three replicates per time point. (E) (Left) Bright-field images of iBTOs 1-, 2-, 4- and 6-weeks post-sort from 11-week LPOs on an inverted microscope. (Right) Quantification of iBTO growth from the day of sorting to 6 weeks post-sorting as the number of cells in organoids generated from the same number of starting cells from iBTOs sorted from 4-6 week LPOs (early sorts) or 10-11 week LPOs (late sorts). (F) Organoid-forming efficiency assay of iBTOs sorted from 4-6 week LPOs (early sort) or 10-11 week LPOs (late sort). Organoid-forming efficiency was measured as the number of cysts formed during the 2 weeks after iBTOs were digested to a single cell suspension and re-plated in Matrigel at 1000 or 2500 cells per μl.

Although LPOs at every time point contain a proliferating population of iBTPs, given the large population and persistence of hindgut lineages, we aimed to isolate NKX2-1-EGFP^+^/CPM^+^ cells to generate higher purities of induced bud tip progenitor organoid (iBTO) cultures. iBTPs were isolated via FACS with CPM and NKX2-1-EGFP (or CPM only for non-reporter cell lines) and were replated in Matrigel at 5000 cells/µl in 3F medium to form iBTOs (Fig. 3A). After sorted cells formed into organoids, iBTOs were passaged by whole passaging approximately every 2 weeks. The NKX2-1-EGFP reporter line showed uniform expression of NKX2-1-EGFP in iBTOs (Fig. 3B), and immunofluorescence of paraffin wax-embedded sections showed iBTOs contain a near-homogenous population of cells expressing BTP markers CPM, SOX9 and SOX2 (Fig. 3C). Interestingly, the age of LPOs at the time of sorting was a crucial determinant of the ability of iBTOs to maintain NKX2-1 and CPM expression. For example, iBTOs generated from <6-week LPOs maintained 32% NKX2-1-EGFP^+^/CPM^+^ cells when re-sorted 7 weeks later (Fig. 3D). In contrast, iBTOs from >6-week LPOs maintained 80% NKX2-1-EGFP^+^/CPM^+^ cells when re-sorted 8 weeks later (Fig. 3D). This suggests that iBTPs undergo increasing commitment to a bud tip progenitor identity as they are maintained in 3F medium at the LPO stage.

To assess iBTO expansion, we evaluated the growth rate of iBTOs from the day iBTPs were isolated from LPOs to 6 weeks of growth as iBTOs. Small cysts could be detected by 1 week of culture, which grew in circumference by 2 weeks (Fig. 3E). By 4 weeks of culture, cysts took on a more complex, branched phenotype, which increased in complexity by 6 weeks (Fig. 3E). Growth of iBTOs sorted from 4- to 6-week LPOs (early timepoints) or 10- to 11-week LPOs (late timepoints) was quantified by counting the number of cells in organoids generated from 40,000 to 75,000 original cells (depending on organoid batch) at 2, 4 and 6 weeks in culture. Overall, a steady increase in cell number was observed for iBTOs sorted from early or late LPOs, with rare batches showing low growth efficiency (Fig. 3E, right). We also measured organoid-forming efficiency of iBTOs by dissociating iBTOs into single cells and replating iBTPs to determine how many cysts formed after 2 weeks. iBTOs from later sorts (10- to 11-week LPOs) had higher organoid-forming efficiency than iBTOs from earlier sorts (4- to 6-week LPOs) (Fig. 3F).

### iPSC-derived bud tip progenitors are transcriptionally similar to primary bud tip progenitor cultures

**Fig. 4.**
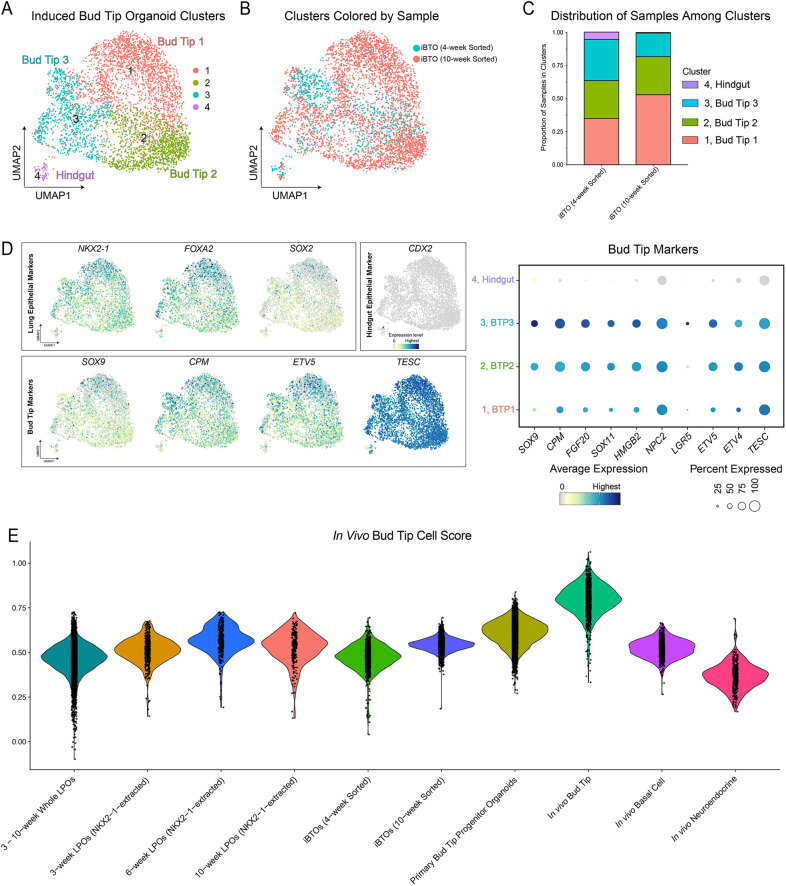
**iPSC-derived bud tip progenitors are transcriptionally similar to human fetal bud tip progenitor cultures.** (A) UMAP cluster plot of scRNA-seq data from iBTOs (*n*=1 biological replicates for iBTOs from 4- and 10-week LPOs). Each dot represents a single cell and cells were computationally clustered based on transcriptional similarities. The plot is colored and numbered by cluster. Cell-type labels for each cluster are based on expression of canonical cell-type markers displayed in the dot plots and feature plots in D and Fig. S4B. (B) UMAP plot corresponding to the iBTO cluster plot in A. Each dot represents a single cell and dots/cell are colored according to the sample from which they came. (C) Stacked bar graph displaying the proportion of cells from each sample in each cluster of the iBTO cluster plot in A. (D) Feature plots and dot plot corresponding to the iBTO cluster plot in A and displaying canonical bud tip progenitor markers (*SOX9*, *CPM*, *ETV5*, *TESC*, *FGF20*, *SOX11*, *HGMB2*, *NPC2*, *LGR5* and *ETV4*) (Miller et al., 2018, 2020; Yamamoto et al., 2020), lung epithelial markers (*NKX2-1*, *FOXA2* and *SOX2*) and hindgut epithelial marker (*CDX2*). The color of each dot in the feature plot indicates log-normalized expression level of the labeled gene in the represented cell. For the dot plot, the dot size represents the percentage of cells expressing the gene in the corresponding cluster, and the dot color indicates log-normalized expression level of the gene. (E) Violin plot displaying an *in vivo* bud tip progenitor cell score, calculated as the average expression of the top 100 enriched genes in *in vivo* bud tip progenitor cells for each sample. Samples include whole LPOs (two 3-week LPOs, two 6-week LPOs and one 10-week LPOs), NXK2-1-extracted cells from 3-, 6- and 10-week LPOs (*n*=2 for 3- and 6-week LPOs, *n*=1 for 10-week LPOs), whole iBTOs (derived from LPOs sorted for NKX2-1^+^/CPM^+^ cells at 4 or 10 weeks, *n*=1 of each), human fetal-derived (primary) bud tip progenitor organoids (14 weeks post-conception) (Miller et al., 2020) and primary *in vivo* tissue (8.5-19 weeks post-conception), including computationally extracted bud tip, basal and neuroendocrine cells (Miller et al., 2020; Hein et al., 2022).

To further interrogate iBTO cellular composition, and to directly compare cells within iBTOs with primary (*in vivo*) bud tip progenitors and primary bud tip progenitor organoids derived from fetal tissue (Miller et al., 2018), we performed scRNA-seq on iBTOs derived from 4- and 10-week LPOs. iBTOs were expanded for 4 weeks in culture post-sorting before scRNA-seq was carried out. Integrated analysis of both datasets resulted in four epithelial clusters (Fig. 4A). Cells in cluster 4 expressed hindgut/intestinal markers (i.e. *CDX2*), which could also be identified by immunofluorescence (Fig. S4A) and represented a small fraction of cells that were predominantly derived from the 4-week sample (Fig. 4B,C). The remaining three clusters (clusters 1, 2 and 3) have enriched expression of *NKX2-1* as well as BTP markers (*SOX9*, *CPM*, *ETV5*, *TESC*, *FGF20*, *SOX11*, *HMGB2*, *NPC2*, *LGR5* and *ETV4*) (Miller et al., 2018, 2020; Hein et al., 2022); however, expression of proliferation genes was variable, suggesting there is some heterogeneity representing different iBTP cell states that is likely driven by the expression of proliferation genes (Fig. 4D, Fig. S4B). Similar to the analysis carried out on LPOs, we quantified *KI67*-expressing cells in each cluster and sample in the iBTO scRNA-seq data (Fig. S4C). All clusters had similar normalized percentages of *KI67*^+^ cells (between 15 and 34%) (Fig. S4C, left panel); however, the contribution of *KI67*^+^ cells to each cluster varied by sample (i.e. timepoint). iBTOs from the 10-week sort contributed more *KI67*^+^ cells to BTP clusters 0 and 1, while iBTOs from the 4-week sort contributed more to the hindgut cluster (cluster 4). Both samples contributed similarly to BTP cluster 3 (Fig. S4C, right panel). Together with the analysis on LPOs (Fig. S2H), this suggests that, although iBTPs are proliferative, the highly proliferative contaminating lineages (i.e. hindgut) can persist in culture despite the sorting strategy if the cultures are sorted too early. This could be attributed to plasticity within cultures during early time points.

To further benchmark transcriptional similarities of iBTPs to *in vivo* BTPs, we re-analyzed published scRNA-seq data from the primary human fetal lung (Miller et al., 2020; Hein et al., 2022) and identified a panel of the top 100 enriched genes expressed in *in vivo* BTPs relative to all other epithelial cell types in the developing lung (see Materials and Methods, Table S1). We used this gene list as a reference to assign an ‘*in vivo* bud tip progenitor cell score’ to each individual cell from the LPOs and iBTOs using published computational methods (Holloway et al., 2020; Hein et al., 2022). For the purposes of this comparison, both whole LPOs and extracted *NKX2-1*^+^ cells from the LPO datasets were included in the analysis. As additional control comparisons, we analyzed published data and included scores for primary BTOs, *in vivo* basal cells and *in vivo* neuroendocrine cells (Miller et al., 2018, 2020; Hein et al., 2022) (Fig. 4E). As expected, *in vivo* BTPs had the highest score (median score=0.80), while *in vivo* neuroendocrine cells had the lowest score (0.37), followed by whole LPOs (0.47). A noteworthy observation is that human primary BTOs had a mean BTP score of 0.62, which was much lower than the *in vivo* BTP score of 0.80, indicating that the *in vitro* culture conditions significantly influence gene expression, as has been observed previously (Miller et al., 2020). Relative to primary BTOs, both *NKX2-1*^+^ cells from LPOs and iBTOs showed high transcriptional similarity; their scores improved with longer time in culture at the LPO stage (0.51 to 0.57 for *NKX2-1^+^* cells from LPOs; 0.48 to 0.54 for iBTOs) (Fig. 4E). To provide additional confidence in this comparison method, we generated a similar *in vivo* basal cell score and *in vivo* neuroendocrine cell score (Table S1) and observed that the expected populations (i.e. *in vivo* basal cells and *in vivo* neuroendocrine cells, respectively) scored the highest relative to all the other data sets analyzed (Fig. S4D).

As an additional analysis, we used published scRNA-seq data from primary human fetal lung epithelium and performed label transfer, which mapped iBTOs to the most similar cell type within the human fetal lung reference dataset (Stuart et al., 2019; Miller et al., 2020; Hein et al., 2022). After clustering the primary epithelial cells (Fig. S4E), iBTOs from LPOs sorted at 4 weeks or 10 weeks largely mapped to four different clusters (Fig. S4F,G). Upon closer investigation into these clusters, three of the clusters (clusters 2, 7 and 9) scored highly for BTP genes (using the BTP cell score) and expressed known markers of BTP cells; the fourth cluster (cluster 6) resembled neuroendocrine progenitor cells (Fig. S4F). Quantification of this mapping revealed that the majority of iBTO cells mapped to two of the three BTP clusters (clusters 2 and 7) whereas only a small subset mapped to the third BTP cluster (cluster 9) and the neuroendocrine cluster (cluster 6) (Fig. S4G). Taken together, these data support the conclusion that iBTOs contain a stable bud tip progenitor cell that shares a high degree of transcriptional similarity with primary bud tip progenitors.

### iBTOs can give rise to airway or alveolar fates

**Fig. 5.**
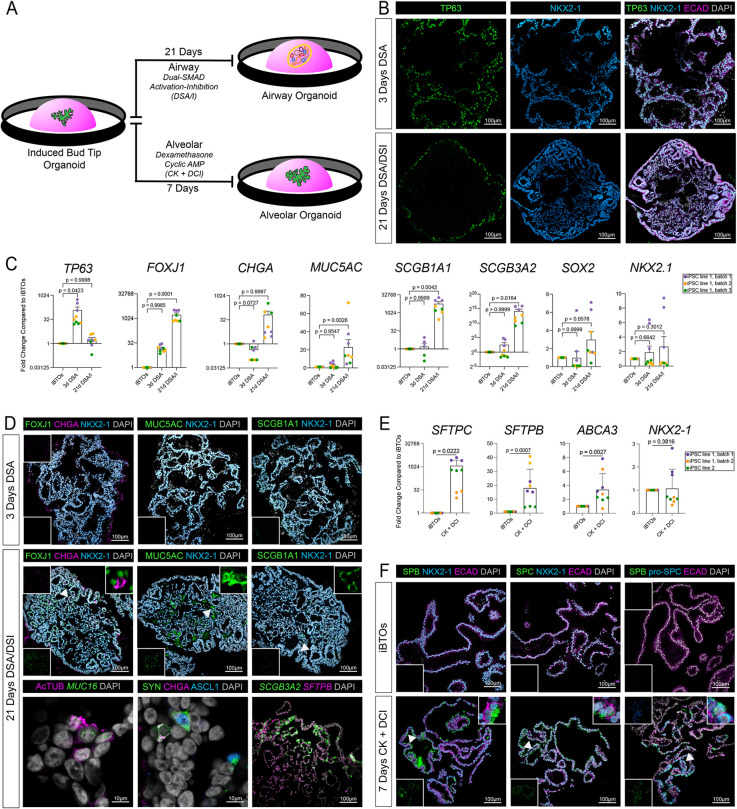
**iBTOs are competent for proximal airway and distal alveolar differentiation.** (A) Schematic displaying the airway (Miller et al., 2020) and alveolar (Jacob et al., 2017) induction protocols from iBTOs. (B) Immunofluorescence staining on paraffin wax-embedded sections for the airway progenitor marker TP63, lung epithelial marker NKX2-1 and general epithelial marker ECAD on iBTOs that have undergone 3 days of dual-SMAD activation (DSA) or 3 days of DSA followed by 18 days of dual-SMAD inactivation (DSI) in the airway induction protocol. (C) qRT-PCR data comparing expression of airway markers *TP63*, *FOXJ1*, *CHGA*, *MUC5AC*, *SCGB1A1*, *SCGB3A2* and *SOX2*, and lung epithelial marker *NKX2-1* in untreated iBTOs or iBTOs that have undergone 3 days of DSA or 3 days of DSA followed by 18 days of DSI. Each colored dot represents a result from an independent experiment using the iPSC17 WT 7B2 line. Data are mean±s.e.m. Statistical tests used were one-way ANOVA followed by Dunnett's multiple comparison test. (D) Fluorescence *in situ* hybridization and/or immunofluorescence staining on paraffin wax-embedded sections for differentiated airway epithelial markers (multiciliated, FOXJ1, AcTUB and *MUC16*; neuroendocrine, CHGA, SYN and ASCL1; goblet, MUC5AC; secretory, *SCGB1A1*, *SCGB3A2* and *SFTPB*) and the lung epithelial marker NKX2-1 on iBTOs that have undergone 3 days DSA or 3 days DSA followed by 18 days of DSI. Insets in the top right corners are zooms of the areas indicated by the arrowheads (DAPI and NKX2-1 channels removed for clarity). Insets in the bottom left or top left corners are single-channel images of non-DAPI or non-NKX2-1 channels, respectively. (E) qRT-PCR data comparing expression of alveolar markers *SFTPC*, *SFTPB* and *ABCA3*, and the lung epithelial marker *NKX2-1* in untreated iBTOs (in 3F medium) or after the alveolar differentiation protocol (7 days CK+DCI). Each colored dot represents a result from an independent experiment with a unique iPSC line or an independent experiment (purple and orange, iPSC17 WT 7B2 LPOs sorted by CPM and NKX2-1-EGFP; green, iPSC line 72.3 LPOs sorted by CPM only). Data are mean±s.e.m. Statistical test used was an unpaired Welch's one-tailed *t*-test. (F) Immunofluorescence staining on paraffin wax-embedded sections for differentiated type II alveolar epithelial markers SFTPB, pro-SFTPC and SFTPC, and the lung epithelial marker NKX2-1 or the general epithelial marker ECAD on iBTOs and iBTOs that have undergone the alveolar differentiation protocol (7 days CK+DCI). Insets in the top right corner are zooms of the area indicated by the arrows. Insets in the bottom left or top left corners are single channel images of non-DAPI and non-NKX2-1 or non-ECAD channels, respectively.

Given that BTPs in the developing lung give rise to airway and alveolar fates, we hypothesized that iBTOs could be guided into airway and alveolar lineages. To test this possibility, we used methods previously developed to efficiently induce lung progenitors into airway (Miller et al., 2020) or alveolar (Jacob et al., 2017) lineages (Fig. 5A). Airway induction involved 3 days of dual-SMAD activation (DSA) followed by 18 days of dual-SMAD inhibition (DSI) and resulted in condensed structures that maintained NKX2-1-EGFP expression and expressed mCherry driven by the TP63 promoter (TP63-mCherry) (Fig. S5A, left column). By immunofluorescence, we observed that TP63 expression was highly induced after 3 days of DSA, as expected; after 18 days of DSI, TP63^+^ cells organized around the perimeter of the organoids (Fig. 5B,C).

We also performed airway induction on unsorted LPOs, hypothesizing that airway differentiation may select for expansion of lung lineages within the LPOs, while suppressing non-lung lineages. NKX2-1-EGFP^+^ and TP63-mCherry^+^ cells were quantified using flow cytometry on both LPOs and iBTOs following the 21-day DSA/DSI protocol or untreated controls (Fig. S5B). Both untreated LPOs and iBTOs contained <1% NKX2-1-EGFP^+^/TP63-mCherry^+^ cells. Only upon treatment with the 21-day DSA/DSI protocol were significant numbers of EGFP^+^/mCherry^+^ cells detected (7% in LPOs, 5% in iBTOs) (Fig. S5B). DSA/DSI-treated iBTOs also maintained a large proportion (71%) of NKX2-1-EGFP^+^/TP63-mCherry^−^ cells, which represented the spectrum of differentiated airway epithelial cell types identified by immunofluorescence and qRT-PCR, including multiciliated, neuroendocrine, goblet and secretory cells (Fig. 5C,D, Fig. S5B). Airway organoids contained AcTUB^+^/*MUC16^+^* ciliated cells (Carraro et al., 2021) (Fig. 5D), and SYN^+^ cells were double positive for ASCL1 (early neuroendocrine marker) or CHGA (late neuroendocrine marker) (Fig. 5D). Treated LPOs contained a small proportion of cells expressing only TP63-mCherry (5%) and a large proportion were negative for both markers (58%) (Fig. S5B,D), suggesting non-lung lineages continue to expand in these conditions.

Alveolar induction consisted of 7 days of treatment with cyclic AMP and dexamethasone (CK+DCI), as has been previously described with the exception of an alternate composition of the base medium (see Materials and Methods) (Jacob et al., 2017). Treatment of iBTOs with CK+DCI resulted in expanded budded structures that maintained NXK2.1-EGFP expression (Fig. S5A, right column). Alveolar type II markers *SFTPB*, *SFTPC* and *ABCA3* were low to undetectable in iBTOs but were highly expressed upon treatment with CK+DCI, as determined by qRT-PCR, and were co-expressed within the same cells, as shown by immunofluorescence for pro-SFTPC, SFTPC, SFTPB and NKX2-1 (Fig. 5E,F). The lung-specific epithelial marker NKX2-1 was robust in both airway and alveolar organoids, while expression of the intestinal epithelial marker CDX2 was not regularly detected (Fig. 5C-F, Fig. S5C). Taken together, differentiation of iBTOs into airway and alveolar cell types with minimal contaminating non-lung cell types supports the observation that iBTOs have similar developmental potential to *in vivo* bud tip progenitors.

## DISCUSSION

In the current work, we demonstrate an optimized *in vitro* model system for differentiating iPSCs into an NKX2-1-expressing bud tip progenitor lineage, which we show can give rise to both airway and alveolar cell types. Several groups, including ours (Dye et al., 2015, 2016; Miller et al., 2018, 2019), have previously characterized protocols that yield lung-like cell types from iPSCs (Wang et al., 2007; Mou et al., 2012; Gotoh et al., 2014; Huang et al., 2014; Konishi et al., 2016; Nikolić et al., 2017; Chen et al., 2017; Hawkins et al., 2017; McCauley et al., 2017; Tamò et al., 2018; de Carvalho et al., 2019; Jacob et al., 2019; Yamamoto et al., 2020; Leibel et al., 2020). Although many of these protocols capture a transient bud tip progenitor-like stage, they focus primarily on deriving more mature lung epithelial lineages, and the induction and maintenance of NKX2-1^+^ bud tip progenitor-like cells from iPSCs has not been previously reported. Furthermore, benchmarking of induced cells using scRNA-seq in order to compare cell types with a human reference atlas has only recently become commonplace (Holloway et al., 2020; Hor et al., 2020; Yu et al., 2020; Hawkins et al., 2021). The method reported here expands upon an improved understanding of crucial signaling events that regulate bud tip progenitor maintenance in the human lung to better mirror this process in a dish (Conway et al., 2020).

Although we demonstrate that optimizing NKX2-1 expression leads to a 100-fold increase when compared with previously reported methods (Dye et al., 2015; Miller et al., 2019), scRNA-seq and whole-mount immunofluorescence analysis of day 10 spheroids revealed that low levels of NKX2-1 are expressed in many cells, whereas a small number of cells expressed high levels of NKX2-1, highlighting the heterogeneity that still exists at this early timepoint. We also observed non-lung lineages that co-emerge in spheroids, possibly owing to the fact that the same signaling pathways play a role during lineage commitment for multiple lineages, while also reflecting the inherent plasticity of newly committed cells. We observed that non-lung lineages, and particularly hindgut lineages, persisted over time in culture. Based on this observation, there is an opportunity to further refine the signaling pathways that are manipulated to expand and maintain NKX2-1^+^ lung lineages; however, this may prove challenging given the redundant use of some pathways (i.e. WNT) in maintaining stem/progenitor cells from both lineages (Pinto and Clevers, 2005; Kapoor et al., 2007; Volckaert and De Langhe, 2015; Chin et al., 2016; Nikolić and Rawlins, 2017; Yan et al., 2017; Miller et al., 2018; Rabata et al., 2020; Aros et al., 2021). Additionally, as mesenchyme was observed by scRNA-seq at the spheroid and LPO stages, how these cells influence epithelial cell fate should be a topic of further exploration.

We observed that hindgut lineages expand after passaging organoids using the standard approach of mechanically shearing them into small fragments. It is currently unclear whether this phenomenon occurs because shearing increases selection for highly proliferative CDX2^+^ cells, if the mechanical stress results in higher levels of apoptosis in one lineage (i.e. lung) compared with the other, or if shearing disrupts the bud tip niche. Thus, although we have optimized the current methods to generate a high proportion of NKX2-1^+^ cells, LPO cultures still exhibited high plasticity that could be triggered through perturbations such as organoid dissociation.

One important observation from our current study is that induced bud tip progenitors exhibited increased lineage commitment with longer time in culture. Isolating NKX2-1-EGFP^+^/CPM^+^ cells prior to 6 weeks resulted in cultures replete with hindgut cells. Although follow-up experiments are required to determine the origin of contaminating hindgut cells, the fact that we still see these cells in purified iBTO cultures suggests a fate switch from a lung bud tip progenitor to a hindgut identity. On the other hand, purifying cells at 6-10 weeks reliably led to the establishment of NKX2-1-EGFP^+^/CPM^+^ iBTOs that expanded and maintained their fate. Whether or not the mechanism underlying the loss of bud tip progenitors caused by sorting LPO cultures too early is the same as that underlying bud tip progenitor loss caused by shearing cultures is an avenue for future research.

We attempted airway differentiation on unsorted LPOs, hypothesizing that the process of differentiation itself may select against gut lineages and expand only lung lineages; unfortunately, non-lung lineages persisted. Given this result, sorting remains an important step for establishing iBTO cultures that can be differentiated into airway or alveolar cell types in the absence of non-lung cell types. Finally, the experiments carried out here relied heavily on an NKX2-1-EGFP reporter; however, our results suggest that this should not limit studies with non-reporter iPSC lines, as we also observed that sorting with CPM alone highly enriched for bud tip progenitors. As we detected some NKX2-1-EGFP^−^/CPM^+^ cells, identifying additional bud tip progenitor cell surface markers to further improve the purity of cultures may be valuable for enhancing purity in non-reporter iPSC cultures.

The use of emerging technologies such as scRNA-seq has provided crucial insight into the heterogeneity and complexity of human tissues that were once difficult to study (Miller et al., 2020; Yu et al., 2020). New information from human tissue has provided both an atlas against which *in vitro* model systems can be benchmarked and a roadmap that can be used to infer transcriptional and signaling mechanisms that control cellular transitions. Here, we used scRNA-seq to benchmark *in vitro* cultures at several stages of differentiation to catalog the induction, emergence and maintenance of lung-fated cells as they acquire a bud tip progenitor fate. One interesting observation from the benchmarking carried out in this study is a significant shift in the transcriptome when comparing primary *in vivo* tissue with *in vitro* grown primary organoids, even when the source of the cells is the same. For example, here, we compared iBTOs with both primary BTOs and *in vivo* fetal bud tip progenitors. We observed a significant shift when comparing fetal lung bud tip progenitors with primary BTOs, indicating that the *in vitro* environment significantly changes the transcriptome of the cell, as has been recently reported (Miller et al., 2020; Alysandratos et al., 2022preprint). With this caveat in mind, iBTOs shared a very similar transcriptome to primary BTOs and a high degree of similarity to *in vivo* primary bud tip progenitors. The cell scoring metric used to compare similarity across samples also suggested that iBTOs became more transcriptionally similar to the *in vivo* bud tip progenitors as they spent more time in culture, supporting the idea that induced bud tip progenitors undergo continued differentiation towards a bud tip progenitor identity as they are maintained in culture.

Functionally, iBTOs also behave like bud tip progenitors. Using methods to differentiate lung progenitor cells into airway or alveolar cell types (Miller et al., 2020; Jacob et al., 2017), we demonstrated that iBTOs robustly differentiate into airway and alveolar cell types. Taken together, the current work describes a robust iPSC-derived bud tip progenitor model for studying human lung epithelial development and differentiation, and uses scRNA-seq to benchmark both on- and off-target cell types present in the cultures. Practically, this model presents a novel opportunity to expand the downstream efforts of lung organoid studies, where iBTOs can be shared to reduce the expertise needed for lung cell generation and ultimately facilitate faster turnaround through iBTO banking. Overall, this study enhances the utility of iPSC-derived lung organoids to interrogate lung development and to study disease and regeneration.

## MATERIALS AND METHODS

### Tissue processing, staining and quantification

All sectioned fluorescent images were taken using a Nikon A1 confocal microscope, an Olympus IX83 inverted fluorescence microscope or an Olympus IX71 inverted fluorescence microscope. Whole-mount fluorescent images were taken on a Nikon X1 Yokogawa Spinning Disk Microscope. Acquisition parameters were kept consistent for images in the same experiment and all post-image processing was performed equally on all images in the same experiment. Images were assembled in Adobe Photoshop CC 2022.

### Tissue processing

Tissue was immediately fixed in 10% neutral buffered formalin (NBF) for 24 h at room temperature on a rocker. Tissue was then washed three times in UltraPure DNase/RNase-Free Distilled Water (Thermo Fisher, 10977015) for 15 min each and then dehydrated in an alcohol series of concentrations diluted in UltraPure DNase/RNase-Free distilled water for 1 h per solution: 25% methanol, 50% methanol, 75% methanol, 100% methanol, 100% ethanol, 70% ethanol. Dehydrated tissue was then processed into paraffin wax blocks using an automated tissue processor (Leica ASP300) with 1 h solution changes. Sections at 4 µm (fluorescence *in situ* hybridization) or 7 µm (immunofluorescence) were cut from paraffin blocks onto charged glass slides (within 1 week of performing fluorescence *in situ* hybridization). For fluorescence *in situ* hybridization, microtome and slides were sprayed with RNase Away (Thermo Fisher, 700511) before sectioning. Slides were baked for 1 h in 60°C dry oven (within 24 h of performing fluorescence *in situ* hybridization). Slides were stored at room temperature in a slide box containing a silica desiccator packet and the seams sealed with Parafilm wrap.

### Immunofluorescence protein staining on paraffin wax sections

Tissue slides were rehydrated in Histo-Clear II (National Diagnostics, HS-202) twice for 5 min each, followed by serial rinses through the following solutions twice for 2-3 min each: 100% ethanol, 95% ethanol, 70% ethanol and 30% ethanol, and finally in double-distilled water (ddH_2_O) twice for 5 min each. Antigen retrieval was performed by steaming slides in 1×sodium citrate buffer [100 mM trisodium citrate (Sigma, S1804), 0.5% Tween 20 (Thermo Fisher, BP337), pH 6.0] for 20 min, followed by cooling and washing quickly twice in ddH_2_O and twice in 1×PBS. Slides were incubated in a humidified chamber at room temperature for 1 h with blocking solution (5% normal donkey serum (Sigma, D9663) in PBS with 0.1% Tween 20). Slides were then incubated in primary antibody diluted in blocking solution at 4°C overnight in a humidified chamber. Next, slides were washed three times in 1×PBS for 5 min each and incubated with secondary antibody with DAPI (1 µg/ml) diluted in blocking solution for 1 h at room temperature in a humidified chamber. Slides were then washed three times in 1×PBS for 5 min each and mounted with ProLong Gold (Thermo Fisher, P36930) and imaged within 2 weeks. Stained slides were stored in the dark at 4°C. All primary and secondary antibody concentrations are listed in Table S2. Immunofluorescence and fluorescence *in situ* hybridization stains were repeated on at least three independent differentiations and representative images are shown.

### Fluorescence *in situ* hybridization

The fluorescence *in situ* hybridization protocol was performed according to the manufacturer's instructions (ACDbio, RNAscope multiplex fluorescent manual) with a 5-min protease treatment and 15-min antigen retrieval. For immunofluorescence co-staining with antibodies, the last step of the fluorescence *in situ* hybridization protocol was omitted and instead the slides were washed once in PBS followed by the immunofluorescence protocol above, starting at the blocking step. A list of probes and reagents can be found in Table S2. Immunofluorescence and fluorescence *in situ* hybridization stains were repeated on at least three independent differentiations and representative images are shown.

### Whole-mount immunofluorescence protein staining

All tips and tubes were coated with 1% BSA in PBS to prevent tissue sticking. 3D cultures (spheroids, LPOs) were dislodged from Matrigel using a P1000 cut tip and transferred to a 1.5 ml microcentrifuge tube. 500 µl of Cell Recovery Solution (Corning, 354253) was added to the tube and the tube was placed on a rocker at 4°C for 45 min to completely dissolve Matrigel. Tube was spun at 100 ***g*** for 5 min, and solution with remaining Matrigel was then removed. Tissue was fixed in 10% NBF overnight at room temperature on a rocker. Tissue then was washed three times for 2 h with 1 ml organoid wash buffer (OWB) (0.1% Triton, 0.2% BSA in 1×PBS) at room temperature on a rocker. Wash times varied (30 min–2 h) depending on tissue size. 1 ml CUBIC-L (TCI Chemicals, T3740) was added to the tube, and the tube was placed on a rocker for 24 h at 37°C. Tissue was then permeabilized for 24 h at 4°C on a rocker with 1 ml permeabilization solution (5% normal donkey serum, 0.5% Triton in 1×PBS). After 24 h, permeabilization solution was removed and 500 µl primary antibody (diluted in OWB) was added overnight at 4°C on a rocker. The next day, tissue was washed three times with 1 ml of OWB, for 2 h each at room temperature. Secondary antibody (500 µl diluted in OWB) was added and incubated overnight at 4°C, wrapped in foil. Tissue was washed again three times with 1 ml OWB at room temperature: first wash for 2 h then 30 min for the remaining washes. Samples were transferred to imaging plate (ThermoFisher, 12-566-70) and then cleared and mounted with 50 µl CUBIC-R (only enough to cover tissue) (TCI Chemicals, T3741). Immunofluorescence and fluorescence *in situ* hybridization stains were repeated on at least three independent differentiations and representative images are shown.

### Cell lines and culture conditions

#### hPSC lines and culture conditions

##### hPSC lines

LPOs were generated from three human induced pluripotent stem cell (iPSC) lines: WTC11 (RRID: CVCL_Y803) was obtained from Bruce Conklin at the University of California San Francisco (USA) (Kreitzer et al., 2013), human iPSC line 72.3 was obtained from Cincinnati Children's Hospital Medical Center (USA) (McCracken et al., 2014) and iPSC17 WT 7B2, which expresses NKX2-1-EGFP and TP63-mCherry, was obtained from the Cystic Fibrosis Foundation (Therapeutics Lab, Lexington, MA, USA) (Crane et al., 2015). In addition to iPSC lines, human embryonic stem cell (hESC) lines were used for definitive endoderm and spheroid optimizations: hESC line H9 (NIH registry no. 0062) was obtained from the WiCell Research Institute and hESC line RUES2-GLR (NIH registry number 0013), expressing SOX2-mCitrine, BRA-mCerulean and SOX17-tdTomato, was obtained from The Rockefeller University (New York, USA) (Martyn et al., 2018). The University of Michigan Human Pluripotent Stem Cell Research Oversight Committee approved all experiments using hESC and iPSC lines. Stem cells were maintained as previously described (Spence et al., 2011) and grown in mTeSR Plus medium (Stem Cell Technologies, 100-0276).

##### NKX2-1-optimized spheroid differentiation protocol

Generation of definitive endoderm (DE) from hPSCs (differentiation days 1–3) was carried out as previously described with slight modifications (D'Amour et al., 2006; McCracken et al., 2011; Spence et al., 2011; Dye et al., 2015). Briefly, 100 ng/ml activin A (R&D Systems, 338-AC) was added in RPMI 1640 medium (Thermo Fisher, 21875034) with increasing concentrations of HyClone defined FBS (dFBS) (Thermo Fisher, SH3007002) on subsequent days (0% day 1, 0.2% day 2 and 2% day 3). 50 ng/ml BMP4 (R&D Systems, 314-BP) was added on day 1. After DE specification, anterior foregut spheroids were generated (differentiation days 4-6) by a 3-day treatment with 500 ng/ml FGF4 (lab purified – see supplementary Materials and Methods), 200 ng/ml noggin (R&D Systems, 6057-NG) and 2 µM CHIR99021 (APExBIO, A3011). All trans retinoic acid (2 µM; Sigma, R2625) was added on the final day of anterior foregut spheroid generation (day 6). On day 7, self-organizing 3D spheroids that had detached from the tissue culture dish were collected with a P200 pipette and transferred into Matrigel (Corning, 354234) as previously described (McCracken et al., 2011; Miller et al., 2019). After the Matrigel had solidified, encapsulating spheroids, they were cultured in medium containing 250 ng/ml WNT3a (R&D Systems, 5036-WN), 500 ng/ml RSPO1 (lab purified – see supplementary Materials and Methods) and 10 ng/ml BMP4 for 3 days to induce NKX2-1^+^ cells (differentiation days 7-9). After DE specification (after day 3), RPMI 1640 medium+2% dFBS was used on all days to dilute growth factors. Fresh media and growth factors were added each day.

##### Growth and maintenance of LPOs

NKX2-1-optimized spheroids were transferred to ‘3 Factor’ (3F) bud tip maintenance medium as previously described (Miller et al., 2018, 2019), including 50 nM all-trans retinoic acid (Sigma, R2625), 10 ng/ml FGF7 (R&D Systems, 251-KG) and 3 µM CHIR99021 (APExBIO, A3011) in serum-free basal medium. Serum-free basal medium consists of DMEM/F12 containing HEPES and L-Glutamine (Corning, 10-092-CV), 100 U/ml penicillin-streptomycin (Thermo Fisher, 15140122), 1× B-27 supplement (Thermo Fisher, 17504044), 1× N-2 supplement (Thermo Fisher, 17502048), 0.05% BSA (Sigma, A9647), 50 µg/ml L-ascorbic acid (Sigma, A4544) and 0.4 µM 1-thioglycerol (Sigma, M1753). LPOs were grown for 3 weeks, then whole passaged or needle or pipette sheared every 2-4 weeks. Whole passaging was achieved by collecting LPOs into a 1.5 ml microcentrifuge tube and gently releasing them from Matrigel using a P1000 cut pipette tip (P200 tip was used for young/small LPOs). LPOs were spun in a microcentrifuge tube, residual media and Matrigel were removed, then LPOs were re-suspended in Matrigel (Corning, 354234) with a P1000 cut pipette tip. Approximately 35 µl droplets of Matrigel were placed into the center of wells of a 24-well tissue culture plate (Thermo Fisher, 12565163), and the plate was inverted and placed in an incubator at 37°C for 20 min. Whole passaging was performed approximately every 2-3 weeks at a ratio of 1:2 (individual LPOs were kept whole but each were given more space, i.e. *n* spheroids were given twice the space to grow) for up to 17 weeks. For LPOs passaged by needle or pipette shearing, LPOs were passed through a 27-gauge needle or P200 pipette, respectively, and embedded in fresh Matrigel as previously described (Miller et al., 2018, 2019, 2020). LPOs were fed with 3F medium every 2-4 days.

##### Growth and maintenance of iBTOs

iBTPs in LPOs grown in 3F medium for 3-17 weeks were isolated by FACS (see below). After collection, iBTPs were centrifuged at 300 ***g*** for 5 min at 4°C and medium was removed. Matrigel (Corning, 354234) was added to the cells at a concentration of 5000 cells/µl Matrigel and 3-20 µl droplets of Matrigel were placed into the center of wells of a 24-well tissue culture plate (Thermo Fisher, 12565163). The plate was inverted and placed in an incubator at 37°C for 20 min. iBTPs were fed every 2-4 days with 3F medium and allowed to reform organoids (iBTOs) for up to 4 weeks. iBTOs were whole passaged as described above every 2-3 weeks after organoid formation.

##### Airway and alveolar differentiations

Airway differentiation was carried out as previously described (Miller et al., 2020). Briefly, iBTOs were exposed to dual-SMAD activation via 100 ng/ml BMP4 (R&D Systems, 314-BP-050) and 100 ng/ml TGFβ1 (R&D Systems, 240-B-002) in 3F medium (described above) for 3 days. On the fourth day, iBTOs were exposed to dual-SMAD inactivation via 1 µM A8301 (APExBIO, 3133), 100 ng/ml noggin (R&D Systems, 6057), 10 µM Y-27632 (APExBIO, B1293) and 500 ng/ml FGF10 (lab purified – see supplementary Materials and Methods) in serum-free basal medium for 18 days (media changed every 3–4 days) with whole passaging as necessary.

Alveolar differentiation was carried out as previously described (Jacob et al., 2017). Briefly, iBTOs were transitioned to alveolar differentiation medium for 7 days (media changed on days 3 and 6). Alveolar differentiation medium consists of a modified serum-free basal medium (SFB-VA) with DMEM/F12 containing HEPES and L-glutamine (Corning, 10-092-CV) supplemented with 1× N-2 supplement (Thermo Fisher, 17502048), 1× B-27 supplement without vitamin A (Thermo Fisher, 12587010), 0.05% BSA (Sigma, A9647), 100 U/ml penicillin-streptomycin (Thermo Fisher, 15140122), 50 µg/ml L-ascorbic acid (Sigma, A9647) and 0.4 µM 1-thioglycerol (Sigma, M6145). On the first day of alveolar differentiation, SFB-VA was supplemented with 10 ng/ml FGF7 (R&D Systems, 251-KG/CF), 3 µM CHIR99021 (APExBIO, A3011), 100 µM 3-Isobutyl-1-methylxanthine (Sigma, I5879), 100 µM 8-bromoadenosine 3′, 5′ -cyclic monophosphate sodium salt (Sigma, B7780) and 50 nM dexamethasone (Sigma, D4802).

##### iBTO growth rate experiments

iBTPs in LPOs grown in 3F medium for 4-6 weeks (early timepoints) or 10-11 weeks (late timepoints) were isolated by FACS (see below). After collection, iBTPs were centrifuged at 300 ***g*** for 5 min at 4°C and media was removed. Matrigel (Corning, 354234) was added to the cells at a concentration of 5000 cells/µl Matrigel and 8-15 µl droplets (droplet size was kept consistent per batch) of Matrigel were placed into the center of wells of a 24-well tissue culture plate (Thermo Fisher, 12565163). The plate was inverted and placed in an incubator at 37°C for 20 min. iBTPs were fed every 2-4 days with 3F medium. At timepoints 2, 4 and 6 weeks post-FACS, iBTOs from one original well of plated iBTPs were collected. iBTOs were removed from Matrigel using a P1000 and/or a P200 pipette tip and vigorously pipetted in a 1.5 ml tube to remove as much Matrigel as possible. Tissue was centrifuged at 300 ***g*** for 5 min at 4°C, then excess media and Matrigel were removed. Tissue was digested to single cells using 250-500 µl TrypLE (Invitrogen, 12605010), depending on pellet size, and incubated at 37°C for 5-15 min, adding mechanical digestion with a pipette every 5 min, until a single cell suspension was reached. Trypsinization was quenched with DMEM/F-12 (Corning, 10-092-CV)+10 µM Y-27632 (APExBIO, B1293). Cells were centrifuged at 300 ***g*** for 5 min at 4°C, then liquid was removed. Cells were resuspended in 100-500 µl 1× PBS+10 µM Y-27632 and counted using a hemocytometer.

##### Organoid forming efficiency assay

After dissociation for iBTO growth rate experiments described above, at the 4-week timepoint, iBTPs were centrifuged at 300 ***g*** for 5 min at 4°C, then media were removed. Matrigel (Corning, 354234) was added to the cells at a concentration of 1000 cells/µl for late timepoints or 25,000 cells/µl for early timepoints (organoids did not form at 1000 cells/µl for early timepoints), and 20 µl droplets of Matrigel were placed into the center of wells of a 24-well tissue culture plate (Thermo Fisher, 12565163). The plate was inverted and placed in an incubator at 37°C for 20 min. iBTPs were fed every 2-4 days with 3F medium. Two weeks later, cultures were imaged for counting the number of cysts. Counting was carried out using the count tool in Adobe Photoshop CC 2022.

#### Culture media, growth factors and small molecules

Additional information for media, growth factors and small molecules, and the in-house expression and purification of human recombinant proteins (FGF4, RSPO1 and FGF10), can be found in the supplementary Materials and Methods.

### Tissue prep for scRNA-seq

All tubes and pipette tips were pre-washed in 1× HBSS with 1% BSA to prevent cell adhesion to the plastic. 3D cultures (spheroids, LPOs, iBTOs) were removed from Matrigel using a P1000 pipette tip and vigorously pipetted in a 1.5 ml microcentrifuge tube to remove as much Matrigel as possible. Tissue was centrifuged at 300 ***g*** for 3 min at 4°C, then excess media and Matrigel were removed. Tissue was digested to single cells using 0.5 ml TrypLE (Invitrogen, 12605010) and incubated at 37°C for 30 min with mechanical digestion with a pipette applied every 10 min. After 30 min, trypsinization was quenched with 1× HBSS+1% BSA. Cells were passed through a 40 µm filter (Bel-Art Flowmi, 136800040) and centrifuged at 300 ***g*** for 3 min at 4°C. Cells were resuspended in 1 ml 1× HBSS+1% BSA and counted using a hemocytometer, centrifuged at 300 ***g*** for 3 min at 4°C, and resuspended to a final concentration of 1100 cells/µl. If samples were planned for combined submission, cells would be cryopreserved in CryoStor CS10 (Biolife Solutions, 210102). If cryopreservation was used, ice-cold CryoStor CS10 solution was added to the cells and mixed thoroughly then transferred to a cryovial. The cells were incubated at 2-8°C for 10 min, cryopreserved in an isopropanol freezing container for 24 h and then transferred to liquid nitrogen. Cells were thawed in a 37°C water bath, washed in DMEM/F12 (Corning, 10-092-CV) with 10% FBS, centrifuged at 300 ***g*** for 5 min, and supernatant was removed. The pellet was then washed in HBSS+1% BSA, then resuspended in 1 ml fresh HBSS+1% BSA, passed through a 40 µm filter (Bel-Art Flowmi, 136800040) and counted using a hemocytometer. Cells were centrifuged at 300 ***g*** for 3 min at 4°C and resuspended to a final concentration of 1100 cells/µl. Approximately 100,000 fresh or thawed cells were put on ice and single cell libraries were immediately prepared at the 10X Chromium at the University of Michigan Sequencing Core with a target of 10,000 cells per sample.

### RNA extraction, cDNA and qRT-PCR

Each analysis includes three biological replicates from three separate differentiation attempts, as well as three technical replicates. mRNA was isolated using the MagMAX-96 Total RNA Isolation Kit (Thermo Fisher, AM1830) (airway and alveolar differentiations) or the PicoPure RNA Isolation Kit (Thermo Fisher, KIT0204) (spheroids). RNA quality and yield were measured on a Nanodrop 2000 spectrophotometer immediately before cDNA synthesis. cDNA synthesis was performed using 100 ng RNA per sample with the SuperScript VILO cDNA Kit (Thermo Fisher, 11754250). qRT-PCR was performed on a Step One Plus Real-Time PCR System (Thermo Fisher, 42765592R) using QuantiTect SYBR Green PCR Kit (Qiagen, 204145). Primer sequences can be found in Table S2. Gene expression as a measure of arbitrary units was calculated relative to GAPDH using the following equation:

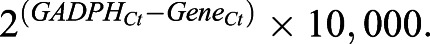


### Bioinformatics/scRNA-seq

#### Overview

To visualize distinct cell populations within the single cell RNA sequencing dataset, we employed the recommended workflow outlined by the Seurat 4.0 R package (Hao et al., 2021). This pipeline includes the following steps: filtering cells for quality control by applying the SCTransform technique (Hafemeister and Satija, 2019) in place of traditional log normalization; variable gene selection and scaling; identifying anchors and integrating if multiple single cell RNA samples are involved (Stuart et al., 2019); reducing dimensionality with principal component analysis (PCA) and uniform manifold approximation and projection (UMAP) (McInnes et al., 2018 preprint; Becht et al., 2019); clustering by either the Louvain algorithm (Blondel et al., 2008) or the Leiden algorithm (Traag et al., 2019); and log normalization on RNA assay for final visualization and for differential gene expression analysis.

#### Sequencing data and processing FASTQ reads into gene expression matrices

All single cell RNA sequencing was performed at the University of Michigan Advanced Genomics Core with an Illumina Novaseq 6000. The 10X Genomics Cell Ranger pipeline was used to process raw Illumina base calls (BCLs) into gene expression matrices. BCL files were demultiplexed to trim adaptor sequences and unique molecular identifiers (UMIs) from reads. Each sample was then aligned to the human reference genome (hg19) to create a filtered feature bar code matrix that contains only the detectable genes for each sample.

#### Quality control

To ensure quality of the data, all samples were filtered to remove cells expressing too few or too many genes (Fig. 1F,G, Fig. S1G and Fig. S4E-G, <500 and >8000; Fig. 2G, Fig. S2E-H and Fig. S3A, <500 and >7000; Fig. 4A-D and Fig. S4B,C, <200 and >9500), with too low or too high UMI counts (Fig. 1F,G and Fig. S1G, <500, >50,000; Fig. 2G, Fig. S2E-H and Fig. S3A, <500 and >50,000; Fig. 4A-D and Fig. S4B-C, <200 and >50,000; Fig. S4E-G, <500 and >60,000) or a fraction of mitochondrial genes greater than 0.1. Following the above steps, a total of 1067 cells and 36,601 genes (Fig. 1F,G and Fig. S1G), 9133 cells and 36,601 genes (Fig. 2G, Fig. S2E-H and Fig. S3A), 4334 cells and 36,602 genes (Fig. 4A-D and Fig. S4B,C); and 10,888 cells and 34,738 genes (Fig. S4E-G) were kept for downstream analysis and visualization.

#### SCTransform and integration

Seurat's SCTransform method allows efficient pre-processing, normalization and variance stabilization of molecular count data from scRNA-seq samples. Running this algorithm will reveal a model of technical noise in the scRNA-seq data through ‘regularized negative binomial regression’, the residuals of which are returned as the SCTransform-normalized values that can be used for further downstream analysis such as dimension reduction. During the SCTransform process, we also chose to regress out a confounding source of variation: mitochondrial mapping percentage. When dealing with one sample (Fig. 1F,G and Fig. S1G), there is no batch effect. But when multiple samples are present (Fig. 2G, Fig. S2E-H, Fig. S3A, Fig. 4A-D, Fig. S4B,C and Fig. S4E-G), we have noticed certain amounts of batch effects when clustering data, owing to technical artifacts such as timing of data acquisition or differences in dissociation protocol. To mitigate these effects, we chose to follow Seurat's integration workflow due to its optimal efficiency in harmonizing large datasets. The two methods used are integration on SCTransform-normalized datasets (Fig. 2G, Fig. S2E-H, Fig. S3A, Fig. 4A-D and Fig. S4B,C) and integration on Log-normalized datasets (Fig. S4E-G). After completion of such batch correction, cell clustering should no longer be driven by technical artifacts.

#### Dimension reduction and clustering

Principal component analysis (PCA) was conducted on the corrected expression matrix as follows. Using the top principal components, a neighborhood graph was calculated for the 20 nearest neighbors (Fig. 1F,G and Fig. S1G, 20 principal components) or the 30 nearest neighbors (Fig. 2G, Fig. S2E-H, Fig. S3A, Fig. 4A-D, Fig. S4B,C and Fig. S4E-G, 30 principal components). The UMAP algorithm was then applied for visualization in two dimensions. Using the Leiden algorithm, clusters were identified with a resolution of 0.2 (Fig. 1F,G, Fig. S1G, Fig. 4A-D, Fig. S4B,C and Fig. S4E-G). Using the Louvain algorithm, clusters were identified with a resolution of 0.08 (Fig. 2G, Fig. S2E-H and Fig. S3A).

#### Cluster annotation

Using canonically expressed gene markers, the general cell identity of each cluster was annotated. Cell identities (with markers) include epithelial (*EPCAM*, *KRT18*, *KRT8* and *CLDN6*), mesenchymal (*POSTN*, *DCN*, *COL1A2* and *COL3A1*), neuronal (*S100B*, *STMN2*, *ELAVL4* and *ASCL1*), endothelial (*ESAM*, *CDH5*, *CLDN5* and *KDR*), proliferative (*MKI67*, *TOP2A* and *CDK1*), primordial germ cell (*POU5F1*, *NANOG*, *TBXT*, *NANOS3* and *TFAP2C*), and foregut mesodermal (*ISL1*, *HAND1*, *BMP4*, *FOXF1* and *LEF1*).

#### Cell scoring

Gene lists for cell scoring (Fig. 4E/Fig. S4D) are found in Table S1 and application of cell scoring strategy is as previously described (Holloway et al., 2020; Hein et al., 2022). Briefly, cells were scored based on expression of a set of 100 marker genes per cell type. Gene lists were compiled by analyzing previously published data from human fetal lung (Miller et al., 2020; Hein et al., 2022). Clusters were first identified by major cell classes (epithelium, mesenchyme, neuronal, endothelium and immune) then the epithelium was sub-clustered to identify bud tip progenitor, basal and neuroendocrine cell clusters by visualizing canonical marker gene expression for each respective cell type. In the case of bud tip progenitor and basal cells, clusters were again sub-clustered to identify the clusters with enriched bud tip progenitor or basal cell marker expression, respectively. Setting the rest of the epithelial cell population as the comparison group, the top 100 differentially expressed genes from bud tip progenitor, basal or neuroendocrine cell clusters were defined as the gene sets for cell scoring. See Table S1 for gene lists. After obtaining the scaled expression values for the dataset, scores for each cell were calculated with the AddModuleScore function of Seurat. Cell scores were visualized by violin plots or feature plots.

#### Normalization for visualization and differential gene expression

As recommended by Seurat developers, we employed the method of log normalization on the standard RNA assay for graphing dot plots and feature plots, and conducting DGEs. Expression matrix read counts per cell were normalized by the total expression, multiplied by a scale factor of 10,000, and finally log transformed. For the differential gene expression testing, we tested only features that are, first, detected in a minimum fraction of 0.25 in either of the two cell populations and, second, show at least 0.25-fold difference in log-scale between the two cell populations on average.

#### Quantification of *KI67*^+^ cells

First, the cells in our LPO and iBTO data were grouped by their sample origin or cluster assignment. Then, within each group, we established the frequency table of *KI67* expression based on *KI67* RNA count of an individual cell, and all cells with expression>0 were quantified as *KI67*^+^. To calculate the contribution of each cluster to *KI67*^+^ cells in the data, the number of *KI67*^+^ cells in each cluster was divided by the total number of cells in the given cluster (‘normalized value’) and then calculated as a percentage of the total normalized values of all the clusters. To calculate the contribution of each sample to *KI67*^+^ cells per cluster, a ‘normalization ratio’ for each cluster per sample was created by dividing the number of cells each sample contributed to each cluster by the total number of cells in the given sample. The normalization ratio was then used to recalculate a ‘normalized’ number of *KI67*^+^ cells in each cluster by multiplying the ratio by the number of *KI67*^+^ cells in each cluster by sample. The percentage of contribution of each sample to *KI67*^+^ cells of a given cluster was then calculated by dividing the number of normalized *KI67*^+^ cells from each sample in each cluster by the total number of normalized *KI67*^+^ cells in the given cluster.

#### Label transfer and UMAP projection

Seurat's single cell reference mapping pipeline allows efficient projection between scRNA-seq datasets (Stuart et al., 2019). The log-normalized integration-prepared primary fetal lung epithelial data (Fig. S4E) were set as reference, while the log-normalized PCA-prepared iBTOs samples were the query data in our analysis. Subsequently, a set of anchors between the reference and query objects were obtained based on PCA projection and were later used to transfer the reference UMAP structure and cluster annotation. Finally, visualization of query data was achieved based on its predicted UMAP representation and cluster labels (Fig. S4F,G).

### Quantification and statistical analysis

Graphs and statistical analysis for qRT-PCR and FACS quantification were performed in GraphPad Prism Software. See figure legends for the number of replicates used, statistical test performed and the *P*-values used to determine the significance for each analysis.

### Fluorescence-activated cell sorting and flow cytometry

#### hPSC flow cytometry

1 ml Accutase (Sigma, A6964) was added to each well of hPSC cultures in a 6-well plate and incubated at 37°C for 5-10 min, until cells detached. An equal volume of mTeSR Plus medium (StemCell Technologies, 100-0276) with 10 µM Y-27632 (APExBIO, B1293) was added to cells and cells were dissociated mechanically by pipetting with a P1000 pipette twice then centrifuged at 300 ***g*** for 5 min at 4°C. Excess medium was removed and cells were resuspended in fluorescence-activated cell sorting (FACS) buffer (1× PBS containing 2% BSA, 10 µM Y-27632 and 100 U/ml penicillin-streptomycin). Cells were passed through a 70 µm cell strainer, pre-coated with FACS buffer, and centrifuged at 300 ***g*** for 5 min at 4°C. Cells were resuspended in 1 ml FACS buffer and transferred to 5 ml FACS tubes (Corning, 352063). DAPI (0.2 µg/ml) was added to respective tubes. Flow cytometry was performed using a Bio Rad Ze5#3 and accompanying software.

#### 3D culture sorting

3D cultures (for LPOs, iBTOs and airway organoids) were removed from Matrigel using a P1000 pipette tip and vigorously pipetted in a 15 ml conical tube to remove as much Matrigel as possible. Tissue was centrifuged at 300 ***g*** for 3 min at 4°C, then excess media and Matrigel were removed. Tissue was digested to single cells using 2-4 ml TrypLE (Invitrogen, 12605010), depending on pellet size and incubated at 37°C for 30 min, adding mechanical digestion with a pipette every 10 min. After 15 min, DNase I (Qiagen, 79254) was added to the digestion at 7.5 µl/ml TrypLE. After 30 min, trypsinization was quenched with DMEM/F-12 (Corning, 10-092-CV)+10 µM Y-27632 (APExBIO, B1293). Cells were passed through a 70 µm cell strainer, pre-coated with DMEM/F-12+10 µM Y-27632 and centrifuged at 500 ***g*** for 5 min at 4°C. Cells were resuspended in 4 ml FACS buffer (2% BSA, 10 µM Y-27632, 100 U/ml penicillin-streptomycin) and transferred into 5 ml FACS tubes (Corning, 352063). Cells were centrifuged again at 300 ***g*** for 3 min at 4°C, then resuspended in 1 ml FACS buffer and counted. 10^5^ cells were placed into new FACS tubes for all controls (no antibody, DAPI only and individual antibodies/fluorophores) and all remaining cells were centrifuged and resuspended in FACS buffer at a concentration of 10^6^ cells/100 µl. Primary antibodies were incubated for 30 min on ice. FACS buffer (3 ml) was added to each tube after 30 min and tubes were centrifuged at 300 ***g*** for 3 min at 4°C. Cells were washed again with 3 ml FACS buffer and centrifuged at 300 ***g*** for 3 min at 4°C. Secondary antibodies were incubated for 30 min on ice. FACS buffer (3 ml) was added to each tube after 30 min and tubes were centrifuged at 300 ***g*** for 3 min at 4°C. Cells were washed again with 3 ml FACS buffer and centrifuged at 300 ***g*** for 3 min at 4°C. Cells were resuspended in FACS buffer and 0.2 µg/ml DAPI was added to respective tubes. FACS was performed using a Sony MA900 cell sorter and accompanying software. Cells were collected in 1 ml 3F medium+10 µM Y-27632. All primary and secondary antibody concentrations are listed in Table S2.

## Supplementary Material

Reviewer comments
