# Supplementary material for: Stable iPSC-derived NKX2-1+ lung bud tip progenitor organoids give rise to airway and alveolar cell types
Source: Development. 2022 Aug 30;149(20):dev200693. doi: 10.1242/dev.200693 (PMC9534489; doi:10.1242/dev.200693)
Supplement: Reviewer comments [file develop-149-200693_review_history.pdf]

Stable iPSC-derived NKX2-1+ lung bud tip progenitor organoids give rise to airway and alveolar cell types

Renee F. C. Hein, Ansley S. Conchola, Alexis S. Fine, Zhiwei Xiao, Tristan Frum, Lindy K. Brastrom, Mayowa A. Akinwale, Charlie J. Childs, Yu-Hwai Tsai, Emily M. Holloway, Sha Huang, John Mahoney, Idse Heemskerk and Jason Spence
DOI: 10.1242/dev.200693

Editor: Matthias Lutolf

Review timeline

Original submission:	28 February 2022
Editorial decision:	25 April 2022
First revision received:	5 July 2022
Accepted:	28 July 2022

Original submission

First decision letter

MS ID#: DEVELOP/2022/200693

MS TITLE: Stable iPSC-derived NKX2-1+ Lung Bud Tip Progenitor Organoids Give Rise to Airway and Alveolar Cell Types

AUTHORS: Renee F.C. Hein, Ansley S. Conchola, Alexis S. Fine, Zhiwei Xiao, Tristan Frum, Charlie J. Childs, Yu-Hwai Tsai, Emily M. Holloway, Sha Huang, John Mahoney, and Jason Spence

I have now received all the referees reports on the above manuscript, and have reached a decision. The referees' comments are appended below, or you can access them online: please go to BenchPress and click on the 'Manuscripts with Decisions' queue in the Author Area.

The overall evaluation is positive and we would like to publish a revised manuscript in Development, provided that the referees' comments can be satisfactorily addressed. Please attend to all of the reviewers' comments in your revised manuscript and detail them in your point-by-point response. If you do not agree with any of their criticisms or suggestions explain clearly why this is so. It is not necessary to provide mechanistic insights as suggested by reviewer 2. If it would be helpful, you are welcome to contact us to discuss your revision in greater detail. Please send us a point-by-point response indicating your plans for addressing the referee’s comments, and we will look over this and provide further guidance.

Reviewer 1

Advance summary and potential significance to field

Hein et al., have established improved conditions for both deriving and expanding multipotent lung epithelial progenitors (bud tip progenitors) from iPSCs. This is a significant advance and will allow the future interrogation of fate decisions made by these progenitor cells. The authors provide a series of well-controlled experiments detailing the cell derivation and expansion procedures and proving that the cells are indeed multipotent progenitors with high similarity to the in vivo bud tip

© 2022. Published by The Company of Biologists under the terms of the Creative Commons Attribution License (https://creativecommons.org/licenses/by/4.0/).

progenitor cells. They have done a thorough and careful job – my only disappointment is that I cannot view the accompanying movies (presumably a failure of the manuscript submission system!)

Comments for the author

1. In Fig1E, only n = 1 spheroid was analyzed. The sample size was limited.
And the clusters 1-3 in the UMAP looked not very clearly separated with potential transient states/sub-clusters in between. The heterogeneity in the induced differentiated spheroids might be underestimated. The authors could comment on this.
2. It is surprising in Fig2C,F that different passaging methods resulted in different efficiencies of progenitor derivation/maintenance. And is to the authors' credit that they share these details with the community. Could they comment on how much the lung progenitor organoids expand at each passage when they are not sheared? (Proliferation rates would be helpful if they have them) And how big the organoids can grow before necrosis is observed? How much expansion of cell number is there between passage 3 and passage 17, for example, while the overall % of CPM/GFP+ cells is increasing?
3. Similarly, it is clear from the methods that the bud tip organoids (once they reform after sorting) are also expanded without shearing. Can the authors mention this in the main part of the text? And ideally also comment on how much of an increase in cell number they obtain during passaging iBTOs by this method?
4. For the induced bud tip progenitors, what is the colony forming efficiency, and whether this value changes over culturing time (for example 3 vs 10 weeks) which may indicate the maturity of progenitors? (But maybe this cannot be assessed if the authors never passage the organoids by conventional means.)
5. For Fig4 the airway differentiation tests, it could help to evaluate the efficiency with some quantification data, for example qPCR data as the detection of alveolar markers in Fig4G.

Minor comments:
1. 'By immunofluorescence, we observed that TP63 expression was highly induced after 3 days of DSA, as expected, and after 18 days of DSI, TP63+ cells organized around the outside of the organoids (Fig. 4C, S4B).' The reference here S4B should be S5B.

Reviewer 2

Advance summary and potential significance to field

In their manuscript "Stable iPSC-derived NKX2-1+ Lung Bud Tip Progenitor Organoids Give Rise to Airway and Alveolar Cell Types" Hein et al. build on prior work in the directed differentiation of human iPSCs to lung epithelial lineages and develop a protocol to derive with improved efficiency and expand cells that are molecularly similar to bud-tip cells with alveolar and airway differentiation potential. The work comes from a group who have pioneered lung differentiation protocols, primary fetal lung cultures, and studies of human fetal lung development. The derivation of stable, self-renewing, lung bud tips would be an advance in the field. The use of in vivo comparisons, primary tissue datasets, reporter iPSC lines. and multiple iPSC lines are a strength. There are some issues which limit enthusiasm.

Comments for the author

Problems/limitations:
The major proposed novelty here is the expansion of stable multipotent bud tip progenitors. However there are some limitations:
-Lack of mechanistic insights into the specification and maintenance of bud tip progenitors
-Many of the advances presented here are protocol based and the scientific advances are less apparent
-The characterization of bud tip cells, central to the manuscript could be further strengthened
-While the bud tips cells seem to upregulate SFTPC quite efficiently, quantitative data of airway differentiation capacity suggests it is quite inefficient.

© 2022. Published by The Company of Biologists under the terms of the Creative Commons Attribution License (https://creativecommons.org/licenses/by/4.0/).

Minor issues
-Endoderm induction has been extensively characterized previously. The optimization experiments here are clear and helpful from a protocol perspective but to not seek to understand how mesenchymal lineages influence the specification of lung progenitors. In summary, these findings are mostly protocol optimization.
-The increase in NKX2-1 transcript is not further analyzed in terms of increased specification of lung progenitors (more cells) vs increased expression of NKX2-1 within lung progenitors. It would be informative to address this; number of NKX2-1+ cells in the new vs prior protocol, expression of NKX2-1 within lung progenitors (using either CPM or NKX2-1GFP).
-the 12% NKX2-1GFP+ % seems very low for an efficient protocol
-While the investigators have made reasonable efforts to benchmark the bud tip cells this is a central claim of the manuscript. Given that their derivation was previously described by this group and the methods to expand these cells rely on continuation in the media previously identified. The real novelty is the use of surface markers (previously described for lung progenitors) that enrich for a purer population of bud tip cells and the single-cell RNA-Sequencing benchmarking. Additional analysis of these datasets would be helpful as the module score is not very convincing and would be bolstered my additional assessments.

First revision

Author response to reviewers' comments

Reviewer comments below are **black**
text Our response is in blue text.

Reviewer 1 Advance Summary and Potential Significance to Field:
Hein et al., have established improved conditions for both deriving and expanding multipotent lung epithelial progenitors (bud tip progenitors) from iPSCs. This is a significant advance and will allow the future interrogation of fate decisions made by these progenitor cells. The authors provide a series of well- controlled experiments detailing the cell derivation and expansion procedures and proving that the cells are indeed multipotent progenitors with high similarity to the in vivo bud tip progenitor cells. They have done a thorough and careful job – my only disappointment is that I cannot view the accompanying movies (presumably a failure of the manuscript submission system!)

Reviewer 1 Comments for the Author:
1. Fig1E, only n = 1 spheroid was analyzed. The sample size was limited. And the clusters 1-3 in the UMAP looked not very clearly separated with potential transient states/sub-clusters in between. The heterogeneity in the induced differentiated spheroids might be underestimated. The authors could comment on this.

The scRNA-seq data of spheroids presented in this figure includes one batch (i.e. one differentiation experiment) of spheroids, but a single batch of spheroids often contains 50-100 spheroids, depending on batch-to-batch variability. Therefore, the data presented represents cells from dozens of individual spheroids derived from one differentiation/batch. We have updated the main text and methods to clarify this point.

To address the comment about separation of states/sub-clusters, we re-analyzed the scRNA-seq data at multiple resolutions, and we also visualized the clustering using both UMAP and tSNE methods. In both cases (UMAP and tSNE), we observed a similar phenomenon, namely, that there was not a stark spatial separation of clusters. When we applied a lower clustering resolution the structure of the UMAP did not change, but the number of clusters was reduced to two. We interpreted this data to suggest that the main clusters are still quite similar to each other. The exception to this observation are the primordial germ-like cells, which form a distinct cluster, suggesting these are quite different. On the other hand, when a higher resolution is applied, we observed 8 clusters; however, when comparing gene enrichment lists from each of these clusters, we could not detect meaningful differences between many of the

© 2022. Published by The Company of Biologists under the terms of the Creative Commons Attribution License (https://creativecommons.org/licenses/by/4.0/).

clusters. As the data is shown (5 clusters), we can detect small but meaningful differences among clusters 1-3, such that some cells appear more foregut-fated and some appear more hindgut-fated. Therefore, we have chosen to leave the clustering as we had originally presented it because we believe it is the most accurate representation of spheroid heterogeneity given the tools that we have to analyze this question.

We are happy to provide the analysis described above to the reviewer upon their request.

2. It is surprising in Fig 2C,F that different passaging methods resulted in different efficiencies of progenitor derivation/maintenance. And is to the authors' credit that they share these details with the community. Could they comment on how much the lung progenitor organoids expand at each passage when they are not sheared? (Proliferation rates would be helpful if they have them) And how big the organoids can grow before necrosis is observed? How much expansion of cell number is there between passage 3 and passage 17, for example, while the overall % of CPM/GFP+ cells is increasing?

Thank you for this question. We have addressed this comment in several ways, described below:

First, we have added details to the methods explaining that whole passaged LPOs were replated at a density of 1:2 (individual LPOs were kept whole but each were given more space, i.e., n spheroids were given 2x the space to grow) approximately every 2 weeks throughout the entire 3- 17 weeks of culture.

Second, to interrogate proliferation rates of LPOs, we analyzed proliferation in the scRNA-seq data of 3-, 6-, and 10-week LPOs by identifying the $KI67^+$ cells in every cluster within the LPO data. This data showed a non-equivalent distribution of $KI67^+$ cells among each cluster and sample.
This data is discussed in the second section of the results and can be found in Figure S2H.

To address cell death in LPOs, we performed both H&E and IF stains of cleaved caspase 3 on 3-, 10-, and 17-week LPOs. This data showed that cell death was present at each stage of LPO growth, but that the localization of cleaved caspase 3 staining changed from individual cells to luminal regions. Luminal staining is interpreted to be the result of cells that are sloughed off into the lumen. Cleaved caspase 3 staining was not correlated with areas of CDX2 expression, indicating that death of non-lung cell types does not likely influence bud tip progenitor expansion over time. This data can be found in Figure S2I.

3. Similarly, it is clear from the methods that the bud tip organoids (once they reform after sorting) are also expanded without shearing. Can the authors mention this in the main part of the text? And ideally also comment on how much of an increase in cell number they obtain during passaging iBTOs by this method?

The reviewer raises an important point, because cell number and cell expansion will be important for future efforts to scale-up iBTOs. To clarify the method of iBTO expansion, we have added text to state that iBTOs were passaged using the "whole passaging" method.

To address growth rates of iBTOs, we sorted bud tip cells from 4 - 6-week (early timepoint) and 10 - 11-week (late timepoint) LPOs, seeded the same number of cells into a droplet of Matrigel, allowed them to expand, dissociated them into single cells and counted the resulting number of cells present. We performed this experiment on iBTOs after 2-, 4-, and 6-weeks of growth. We also took matching bright field images to show iBTO growth over time in culture. This data showed similar, steady growth rates for iBTOs whether they were derived from early or late LPOs and can be found in Figure 3E.

4. The induced bud tip progenitors, what is the colony forming efficiency, and whether this value changes over culturing time (for example 3 vs 10 weeks) which may indicate the maturity of progenitors? (But maybe this cannot be assessed if the authors never passage the organoids by conventional means.)

© 2022. Published by The Company of Biologists under the terms of the Creative Commons Attribution License (https://creativecommons.org/licenses/by/4.0/).

To address this comment, we performed colony forming efficiency experiments on iBTOs. We performed this experiment on iBTOs formed from LPOs sorted after 4 - 6-weeks and from LPOs sorted after 10 - 11-weeks as the reviewer suggested. This data showed that iBTOs derived from LPOs sorted at 10 - 11-weeks had better organoid forming efficiency than iBTOs sorted from early LPO. This new data can be found in Figure 3F.

5. For Fig4 the airway differentiation tests, it could help to evaluate the efficiency with some quantification data, for example qPCR data as the detection of alveolar markers in Fig4G.

We appreciate this suggestion. To address it, we used qRT-PCR to evaluate the expression of airway cell type markers on iBTOs in bud tip media (3F), iBTOs subjected to 3 days of dual-SMAD activation (DSA), and iBTOs subjected to 3 days of DSA followed by 18 days of dual-SMAD inactivation (21 days DSA/I). This data supported our immunofluorescence stains and can be found in Figure 5C.

Minor comments:
1.'By immunofluorescence, we observed that TP63 expression was highly induced after 3 days of DSA, as expected, and after 18 days of DSI, TP63+ cells organized around the outside of the organoids (Fig. 4C, S4B).' The reference here S4B should be S5B.

Thank you for your careful reading of the manuscript. We have made the appropriate change.

Reviewer 2 Advance Summary and Potential Significance to Field:
In their manuscript "Stable iPSC-derived NKX2-1+ Lung Bud Tip Progenitor Organoids Give Rise to Airway and Alveolar Cell Types" Hein et al. build on prior work in the directed differentiation of human iPSCs to lung epithelial lineages and develop a protocol to derive with improved efficiency and expand cells that are molecularly similar to bud-tip cells with alveolar and airway differentiation potential. The work comes from a group who have pioneered lung differentiation protocols, primary fetal lung cultures, and studies of human fetal lung development. The derivation of stable, self-renewing, lung bud tips would be an advance in the field. The use of in vivo comparisons, primary tissue datasets, reporter iPSC lines. and multiple iPSC lines are a strength. There are some issues which limit enthusiasm.

Reviewer 2 Comments for the Author:
Problems/limitations: The major proposed novelty here is the expansion of stable multipotent bud tip progenitors. However there are some limitations:

-Lack of mechanistic insights into the specification and maintenance of bud tip progenitors

We agree with the reviewer. Our manuscript was meant to develop a model that was thoroughly characterized and benchmarked using rigorous and unbiased methods. While it does not make mechanistic insights, it provides a solid framework for mechanistic follow-up studies. As suggested by the editor, this manuscript will be considered as a "resource" article.

-Many of the advances presented here are protocol based and the scientific advances are less apparent

We agree with the reviewer that this manuscript significantly advances the methods to generate bud tip progenitor organoids from iPSCs. An iBTO model has not previously been reported and we hope that it helps move the field forward by providing a rigorous and well characterized system that accurately approximates a bona fide human fetal bud tip progenitor. We also feel that our transparency in reporting off-target cell types is an important step forward for the field to fully understand the strengths and limitations of our work.

-The characterization of bud tip cells, central to the manuscript could be further strengthened

Previously, we compared induced bud tip cells to primary *in vivo* bud tip cells using a cell scoring method that used the top 100 genes representing *in vivo* bud tip cells from scRNA-seq

© 2022. Published by The Company of Biologists under the terms of the Creative Commons Attribution License (https://creativecommons.org/licenses/by/4.0/).

data. We have now added new data to further characterize induced bud tip progenitors using an approach called label transfer to show which *in vivo* cells induced bud tip cells most closely resemble. This data is explained in the last paragraph of the second-to-last results section and can be found in Figure S4E-G. We have further shown individual bud tip marker genes using scRNA-seq and by immunofluorescence. We feel that computational approaches relying on large gene sets (i.e., cell scoring), label transfer approaches, and showing individual marker genes thoroughly characterizes bud tip cells.

-While the bud tips cells seem to upregulate SFTPC quite efficiently, quantitative data of airway differentiation capacity suggests it is quite inefficient.

To address this, we used qRT-PCR to evaluate the expression of airway cell type markers on iBTOs in bud tip media (3F), iBTOs subjected to 3 days of dual-SMAD activation (DSA), and iBTOs subjected to 3 days of DSA followed by 18 days of dual-SMAD inactivation (21 days DSA/I). This data confirmed our immunofluorescence stains and can be found in Figure 5C.

Minor issues
-Endoderm induction has been extensively characterized previously. The optimization experiments here are clear and helpful from a protocol perspective but to not seek to understand how mesenchymal lineages influence the specification of lung progenitors. In summary, these findings are mostly protocol optimization.

How the mesenchyme influences bud tip progenitor specification is an insightful question. Although we observe foregut mesoderm-like cells in spheroids and some mesenchymal cells in young LPOs, our media is optimized for the epithelium, and mesenchymal lineages are ultimately depleted from the culture. Additionally, mesenchymal cells present in LPO cultures do not appear lung-specific (when markers are observed in scRNA-seq data). Nevertheless, this is an important question that merits follow-up, and as such, we have included mention of this in the discussion section.

-The increase in NKX2-1 transcript is not further analyzed in terms of increased specification of lung progenitors (more cells) vs increased expression of NKX2-1 within lung progenitors. It would be informative to address this; number of NKX2-1+ cells in the new vs prior protocol, expression of NKX2-1 within lung progenitors (using either CPM or NKX2-1GFP).

Figure 2F shows quantitative FACS data and Figure S2F shows quantitative scRNA-seq data on the number of NKX2-1$^+$ cells in LPOs at different timepoints. Both pieces of data show that the number of NKX2-1$^+$ cells increase over time. Because both methods filter out dead cells, only live cells are quantified, so we can be certain that the number of NKX2-1$^+$ cells increase over time, rather than the level of NKX2.1 expression in individual cells.

-the 12% NKX2-1GFP+ % seems very low for an efficient protocol

We agree that 12% of NKX2-1$^+$ cells is low. This refers to the 3-week culture time point. As we show in detail, induction of NKX2-1$^+$ cells increased as time progressed, and, sorted bud tip progenitors from later time points were able to be better maintained. We do not specifically claim that the 3-week time point is efficient, and we have carefully read and edited the text to ensure this point is not misconstrued.

-While the investigators have made reasonable efforts to benchmark the bud tip cells this is a central claim of the manuscript. Given that their derivation was previously described by this group and the methods to expand these cells rely on continuation in the media previously identified. The real novelty is the use of surface markers (previously described for lung progenitors) that enrich for a purer population of bud tip cells and the single-cell RNA-Sequencing benchmarking. Additional analysis of these datasets would be helpful as the module score is not very convincing and would be bolstered my additional assessments.

Please see our response to your earlier concern above. We have now added additional analyses.

© 2022. Published by The Company of Biologists under the terms of the Creative Commons Attribution License (https://creativecommons.org/licenses/by/4.0/).

Second decision letter

MS ID#: DEVELOP/2022/200693

MS TITLE: Stable iPSC-derived NKX2-1+ Lung Bud Tip Progenitor Organoids Give Rise to Airway and Alveolar Cell Types

AUTHORS: Renee F.C. Hein, Ansley S. Conchola, Alexis S. Fine, Zhiwei Xiao, Tristan Frum, Lindy K. Brastrom, Mayowa A. Akinwale, Charlie J. Childs, Yu-Hwai Tsai, Emily M. Holloway, Sha Huang, John Mahoney, Idse Heemskerk, and Jason Spence
ARTICLE TYPE: Research Article

I am happy to tell you that your manuscript has been accepted for publication in Development, pending our standard ethics checks. Please address the minor points raised by reviewer 2.

Reviewer 1

Advance summary and potential significance to field

The authors have done an excellent job of revising the manuscript and fully address all of our concerns.

Comments for the author

N/A

Reviewer 2

Advance summary and potential significance to field

Hein et al. report methods to generate lung bud tip organoids from iPSCs that can be maintained in culture for long periods while maintaining phenotype and also display airway/alveolar competence. This is an advance for iPSC technology in the lung as it improves the scale of experiments that can be performed and offers a platform to study in the program of the developing human lung.

Comments for the author

I have reviewed revised manuscript from Hein et al. As a resource article, the concerns regarding mechanistic insights as a Development research article, are mitigated. Additional data characterizing induced lung bud tip cells are included and strengthen the conclusions. Quantification of a panel airway markers in the airway protocol by RT-PCR are now included which also strengthen the conclusion of airway competence.
Regarding my comment about the increase in NKX2-1 transcript achieved in the new protocol (and described in Figure 1) and the suggestion that quantifying the percentage of NKX2-1+ cells across cell lines and differentiations would be helpful: the authors cite Figure 2F, S2F which address a later timepoint and does not answer my question. It seems that early in the protocol, the percentage of NKX2-1+ cells is quite low but with time, passaging, and sorting the percentage increases to a high level. As a resource article, I still maintain that more details on how efficient the early stage is (as described in Figure 1) across iPSC lines would be important to know. Many groups have published lung differentiation protocols of varying efficiencies and there is ongoing controversy over the best strategy, the need for purification strategies (reporters or surface markers) vs protocols that are sufficiently robust, and a lack of comparisons between protocols. Based on this, I find the following sentence as it appears in the abstract somewhat misleading: “Building on prior work we optimized a directed differentiation paradigm to generate spheroids with robust NKX2-1 expression”. The term “robust” is also used in the first results section. The discussion section is balanced and clear. I suggest including the data on NKX2-1 efficiencies early in the protocol if available (Figure 1, day 10-13). As a resource article it is important for readers to know this data. If not available, then adjusting the language noted above is recommended. Otherwise, I am satisfied that the authors have addressed my concerns and the ability to maintain bud tip organoids in culture is an exciting advance and I congratulate them.

© 2022. Published by The Company of Biologists under the terms of the Creative Commons Attribution License (https://creativecommons.org/licenses/by/4.0/).
